# Devonian–Carboniferous extension and Eurekan inversion along an inherited WNW–ESE-striking fault system in Billefjorden, Svalbard

**DOI:** 10.12688/openreseurope.15936.1

**Published:** 2023-08-04

**Authors:** Jean-Baptiste P. Koehl, Lis Allaart, Riko Noormets

**Affiliations:** 1 Department of Earth and Planetary Sciences, McGill University, Montreal, Québec, H3A 0E8, Canada; 2 Department of Geosciences, University i Oslo, Oslo, Oslo, 0371, Norway; 3 Arctic Geology, University Centre in Svalbard AS, Longyearbyen, Svalbard, 9171, Norway; 4 Department of Biology—Microbiology, Aarhus University, Aarhus C, Denmark

**Keywords:** Svalbard, Billefjorden, Devonian, décollement, Timanian, Svalbardian, Eurekan, Cenozoic

## Abstract

**Background:** The Billefjorden area in central Spitsbergen hosts thick Lower–lowermost Upper Devonian, late–post-Caledonian collapse deposits presumably deformed during the Late Devonian Svalbardian Orogeny. These rocks are juxtaposed against Proterozoic basement rocks along the Billefjorden Fault Zone and are overlain by uppermost Devonian–early Permian deposits of the Billefjorden Trough, a N–S-trending Carboniferous rift basin bounded by the Billefjorden Fault Zone.

**Methods:** We interpreted seismic reflection (also depth-converted), bathymetric, and exploration well data.

**Results:** The data show abundant Early Devonian, WNW–ESE-striking (oblique-slip) normal faults segmenting the Billefjorden Trough, and a gradual decrease in tectonic activity from the Early Devonian (collapse phase) to early Permian (post-rift phase). Early Devonian–Middle Pennsylvanian WNW–ESE-striking faults were mildly reactivated and overprinted and accommodated strain partitioning and decoupling in the early Cenozoic. This resulted in intense deformation of Lower Devonian sedimentary rocks and in the formation of bedding-parallel décollements, e.g., between the Lower Devonian Wood Bay and the uppermost Pennsylvanian–lowermost Permian Wordiekammen formations. This suggests that intense deformation within Devonian rocks in Dickson Land can be explained by Eurekan deformation alone. Eurekan deformation also resulted in the formation of WNW–ESE- and N–S- to NNE–SSW-trending, kilometer-wide, open folds such as the Petuniabukta Syncline, and in inversion and/or overprinting of Early Devonian to Early Pennsylvanian normal faults by sinistral-reverse Eurekan thrusts. WNW–ESE-striking faults merge at depth with similarly trending and dipping ductile shear zone fabrics in Proterozoic basement rocks, which likely formed during the Timanian Orogeny.

**Conclusions:** A NNE-dipping shear zone, which is part of a large system of Timanian thrusts in the Barents Sea, controlled the formation of WNW–ESE-striking Devonian–Mississippian normal faults and syn-tectonic sedimentary rocks in Billefjorden. Eurekan strain partitioning and decoupling suggest that the Svalbardian Orogeny did not occur in Svalbard.

## Introduction

The Svalbard Archipelago experienced a complex series of tectonic events during its geological evolution, including the latest Neoproterozoic Timanian Orogeny ( Faehnrich *et al*., 2020; Koehl, 2019; Koehl, 2020a; Koehl *et al*., 2022a; Majka *et al*., 2008; Majka *et al*., 2012; Majka *et al*., 2014; Mazur *et al*., 2009), early Paleozoic Caledonian Orogeny ( Gee *et al*., 1992; Gee *et al*., 1994; Harland *et al*., 1966; Witt-Nilsson *et al*., 1998), Devonian late- to post-orogenic extension ( Braathen *et al*., 2018; Braathen *et al*., 2020; Friend *et al*., 1997; Maher *et al*., 2022; Manby & Lyberis, 1992), latest Devonian Svalbardian contraction ( Dallmann & Piepjohn, 2020; Harland *et al*., 1974; Piepjohn *et al*., 1997; Vogt, 1938), Pennsylvanian to early Permian rifting ( Braathen *et al*., 2011; Cutbill & Challinor, 1965; Johannessen & Steel, 1992), and early Cenozoic Eurekan deformation ( Dallmann *et al*., 1993; Harland, 1969; Harland & Horsfield, 1974; Maher *et al*., 1986). Apart from the Timanian Orogeny, these tectonic events contributed to form a prominent N–S-trending structural grain on the island of Spitsbergen. This grain consists of Caledonian foliation, thrusts and shear zones, Devonian normal faults, latest Devonian folds and thrusts, Pennsylvanian to early Permian normal faults and early Cenozoic thrusts, folds, and shear zones. The long-lived N–S trend of the dominant structural grain is thought to have played an important role in the assembly of Svalbard’s three main basement terranes during the early to mid Paleozoic (Caledonian and Svalbardian orogenies), e.g., by accommodating strike-slip movements in a scale of hundreds to thousands of kilometers along brittle faults such as the Billefjorden Fault Zone ( Harland *et al*., 1974; Harland *et al*., 1992). Nevertheless, N–S-trending structures alone, cannot explain all the sedimentary thickness variations within Carboniferous successions, such as the Hultberget Formation (e.g., locally absent in Brucebyen; Cutbill *et al*., 1976) and coal-rich strata of the Billefjorden Group (e.g., thickest in Pyramiden, Sassenfjorden, and Tempelfjorden but almost absent in Yggdrasilkampen; Cutbill *et al*., 1976; Dallmann *et al*., 2004a; Koehl, 2021).

Other structural trends exist in Svalbard, among which the WNW–ESE trend is the most prominent ( Bergh *et al*., 2000; Koehl & Muñoz-Barrera, 2018; McCann, 2000; Piepjohn *et al*., 2001; Saalmann & Thiedig, 2000; Saalmann & Thiedig, 2001). Structures of this trend have, thus far, been poorly studied and their role and implications for the tectonic history of Svalbard are poorly understood. The goal of this paper is to discuss newly identified WNW–ESE-striking structures on seismic and bathymetric data in Billefjorden, e.g., the Adolfbukta and Garmaksla faults, and their influence on well-studied N–S-striking basins and faults, e.g., the northern Spitsbergen Devonian Graben ( Friend *et al*., 1997; Manby & Lyberis, 1992) and the Carboniferous Billefjorden Trough ( Braathen *et al*., 2011; Gjelberg, 1984). The present contribution is part of a large study ( Koehl *et al.*, 2020) aiming at investigating cryptic WNW–ESE-striking structures and fabrics in the Norwegian Arctic. The present contribution focuses on the offshore portion (seismic and bathymetric data) of the Billefjorden area, whereas Koehl *et al.* (2023a) and Koehl *et al.* (2023b) focus on onshore outcrops respectively on the western shore and the eastern shore of the fjord.

## Geological setting

In the latest Neoproterozoic, the Svalbard Archipelago was truncated by several kilometers thick, thousands of kilometers long, dominantly top-SSW thrust systems during the Timanian Orogeny ( Koehl, 2019; Koehl, 2020a; Koehl *et al*., 2022a). In northern and central Spitsbergen, these thrusts are deeply buried, but some crop out in western Spitsbergen, where they were exhumed by subsequent E–W Caledonian and Eurekan contraction (e.g., Vimsodden–Kosibapasset Shear Zone in southwestern Spitsbergen; Faehnrich *et al*., 2020; Majka *et al*., 2008; Majka *et al*., 2012; Majka *et al*., 2014; Mazur *et al*., 2009).

**Figure 1.**
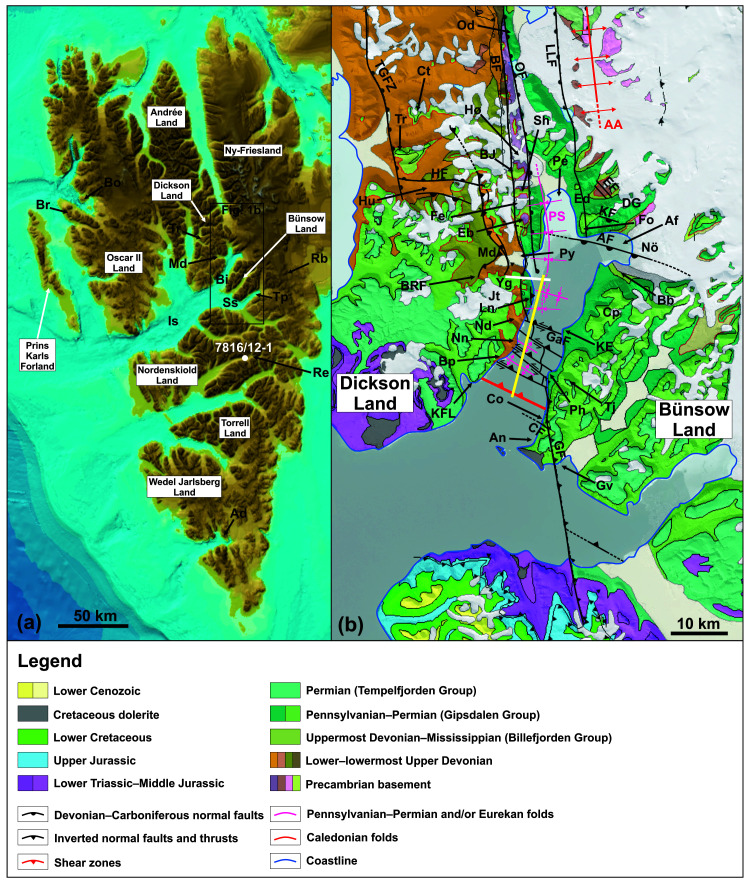
( **A**) Topographic and bathymetric map around Spitsbergen modified after Jakobsson *et al.* (2012) (originally published under CC-BY 4). Abbreviations: Ad – Adriabukta; Bi – Billefjorden; Bo – Blomstrandhalvøya; Br – Brøggerhalvøya; Is – Isfjorden; Md – Mimerdalen; Rb – Rembebreen; Re – Reindalspasset; Ss – Sassenfjorden; Tp – Tempelfjorden; Tr – Triungen; ( **B**) Geological map modified from svalbardkartet.npolar.no showing the main tectono-stratigraphic units and structures in the study area in central Spitsbergen. The yellow line shows the location of the seismic line in Figure 4A–B and the white line the approximate location of the transect shown in Figure 6. Abbreviations: AA – Atomfjella Antiform; AF – Adolfbukta Fault; Af – Adolfbukta; An – Anservika; Bb – Brucebyen; BF – Balliolbreen Fault; BJ – Birger Johnsonfjellet; Bp – Brimerpynten; BRF – Blåvatnet Reverse Fault; CF – Cowantoppen Fault; Co – Cowanodden; Cp – Campbellryggen; Ct – Citadellet; DG – De Geerfjellet; Eb – Elsabreen; Ed – Ebbadalen; EF – Ebbabreen Faults; Fe – Ferdinandbreen; Fo – Fortet; GaF – Garmaksla fault; GF – Gipshuken Fault; Gv – Gipsvika; HF – Hugindalen Fault; Hu – Hugindalen; Hø – Hørbyebreen; Jf – Jotunfonna; KE – Kapp Eckholm; KF – Kampesteindalen Fault; KFL – Kapp Fleur de Lys; LLF – Lemströmfjellet–Løvehovden Fault; Ln – Lykteneset; Md – Mimerdalen; Mu – Mumien; Nd – Nidedalen; Nn – Narveneset; Nö – Nordenskiöldbreen; Od – Odellfjellet; OF – Odelfjellet Fault; Pe – Petuniabukta; PS – Petuniabukta Syncline; Ph – Phantomodden; Py – Pyramiden; Re – Reindalspasset; Sh – Svenbreenhøgda; TGFZ – Triungen–Grønhorgdalen Fault Zone; Tj – Tjosaasfjellet; Tr – Triungen; Yg – Yggdrasilkampen.

In the early Paleozoic, igneous and sedimentary Proterozoic basement rocks in northeastern Spitsbergen ( Figure 1A) were subjected to c. E–W-oriented contraction during the Caledonian Orogeny resulting in the formation of a tens of kilometer wide, gently north-plunging antiform or antiformal thrust stack with well developed N–S-trending foliation ( Gee *et al*., 1992; Gee *et al*., 1994; Witt-Nilsson *et al*., 1998).

In late Silurian to Early Devonian times, late- to post-orogenic extensional collapse initiated, leading to the deposition of several kilometer-thick, reddish sedimentary successions in northern Spitsbergen made up with basal conglomerate units overlain by interbedded sandstones and shales ( Friend & Moody-Stuart, 1972; Gee & Moody-Stuart, 1966; Manby & Lyberis, 1992; Manby *et al*., 1994). These successions were deposited in the hanging wall of low-angle extensional detachments ( Chorowicz, 1992; Roy, 2007; Roy, 2009), some of which accommodated coeval exhumation of basement rocks as metamorphic core complexes ( Braathen *et al*., 2018; Braathen *et al*., 2020; Maher *et al*., 2022). A description of the Devonian sedimentary units in northern and central Spitsbergen is provided in *Extended data*. In Billefjorden ( Figure 1B), Devonian sedimentary rocks are believed to be present west and southwest of the Billefjorden Fault Zone as observed in onshore areas (e.g., Dallmann & Piepjohn, 2020; Piepjohn, 2000).

In the Late Devonian, Spitsbergen is commonly thought to have experienced a short-lived episode of contraction, the Svalbardian Orogeny, during which the Balliolbreen Fault segment of the Billefjorden Fault Zone presumably formed as a top-west reverse fault, juxtaposing Proterozoic basement rocks in the east against post-Caledonian (Devonian) sedimentary rocks in the west ( Bergh *et al*., 2011; Dallmann & Piepjohn, 2020; Harland *et al*., 1974; McCann, 2000; Piepjohn *et al*., 1997; Piepjohn, 2000; Vogt, 1938). The Balliolbreen Fault is thought to continue southwards across Billefjorden as a rectilinear NNW–SSE- to N–S-striking fault ( Bælum & Braathen, 2012). Svalbardian tectonism is thought to have deformed Devonian sedimentary rocks of the Andrée Land Group and Mimerdalen Subgroup intensely in discrete narrow belts in Dickson Land ( Dallmann & Piepjohn, 2020; Piepjohn & Dallmann, 2014). However, competing interpretations based on evidence from aerial photographs and field and seismic data suggest that Svalbardian folds and thrusts may partly have formed during extensional detachment folding in the Early to Middle Devonian ( Chorowicz, 1992; Roy, 2007; Roy, 2009) and/or due to early Cenozoic strain partitioning and decoupling along low-angle detachments and/or décollements, e.g., within weak Devonian to Mississippian shale- and coal-rich sedimentary strata ( Koehl, 2021) and Pennsylvanian to Permian evaporites ( Harland *et al*., 1988; Ringset & Andresen, 1988). In addition, there are many inconsistencies attached to the timing of the Svalbardian event throughout Spitsbergen ( Koehl *et al*., 2022b).

**Figure 2.**
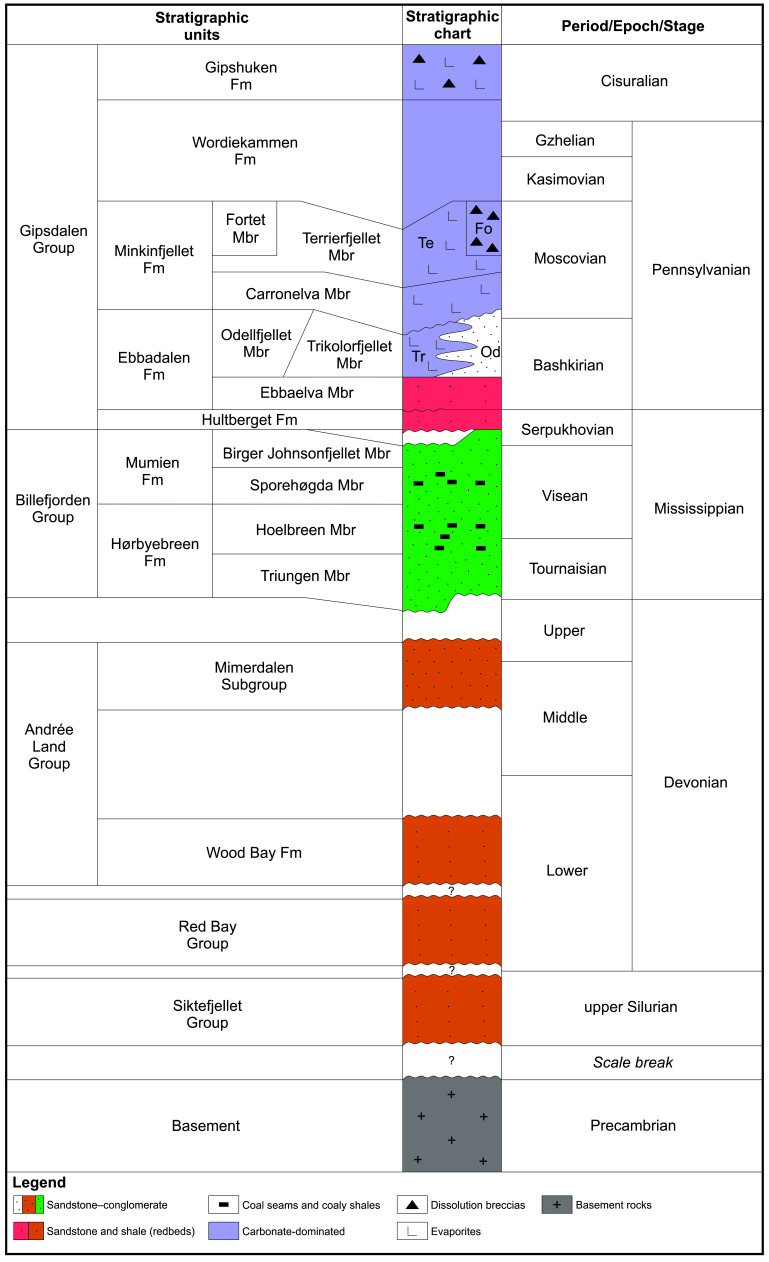
Litho-stratigraphic chart of mid to late Paleozoic (upper Silurian to lower Permian) sedimentary rocks of the Siktefjellet, Red Bay, Andrée Land, Billefjorden, and Gipsdalen groups in central Spitsbergen. The chart is based on descriptions by Aakvik (1981); Braathen *et al.* (2011); Cutbill & Challinor (1965); Cutbill *et al.* (1976); Dallmann *et al.* (1999); Friend *et al.* (1966); Friend & Moody-Stuart (1972); Friend *et al.* (1997); Gee *et al.* (1952); Gee & Moody-Stuart (1966); Gjelberg (1983); Gjelberg (1984); Gjelberg & Steel (1981); Holliday & Cutbill (1972); Johannessen (1980); Johannessen & Steel (1992); Lønøy (1981); Lønøy (1995); McWhae (1953); Murascov & Mokin (1979); Playford (1962), and Dallmann & Piepjohn (2020).

In the latest Devonian to Mississippian ( Lindemann *et al*., 2013; Marshall *et al*., 2015; Playford, 1962; Playford, 1963; Scheibner *et al*., 2012), fluvial, clastic- and coal-rich deposits of the Billefjorden Group ( Figure 2) deposited during a period of tectonic quiescence ( Braathen *et al*., 2011; Smyrak-Sikora *et al*., 2018) or within multiple mini-basins ( Aakvik, 1981; Cutbill & Challinor, 1965; Cutbill *et al*., 1976; Gjelberg, 1984; Koehl & Muñoz-Barrera, 2018; see electronic supplement 1 for description of the stratigraphic units).

In the Earyl to Middle Pennsylvanian, sediment deposition (Gipsdalen Group) was restricted to the Billefjorden Trough and was accompanied by kilometer-scale normal faulting in a shallow marine environment with main depocenter between the Billefjorden Fault Zone and the Lemströmfjellet–Løvehovden Fault ( Braathen *et al*., 2011; Smyrak-Sikora *et al*., 2018; Figure 2). In the latest Middle Pennsylvanian to early Permian, tectonic activity had almost completely ceased and a carbonate platform developed in a shallow sea ( Ahlborn & Stemmerik, 2015; Braathen *et al*., 2011; Cutbill & Challinor, 1965; Gee *et al*., 1952; Keilen, 1992; Maher & Braathen, 2011 see electronic supplement 1 for description of the stratigraphic units).

In the early Cenozoic, the opening of the Labrador Sea and of Baffin Bay between Greenland and Canada ( Chalmers & Pulvercraft, 2001; Oakey & Chalmers, 2012) resulted in an episode of contraction (transpression?) in Svalbard, the Eurekan tectonic event, during which east-verging thrusts formed in the West Spitsbergen Fold-and-Thrust Belt ( Andresen *et al*., 1994; Dallmann *et al*., 1993; Harland, 1969; Harland & Horsfield, 1974; Maher *et al*., 1986) and a thick succession of sediments deposited in the Central Tertiary Basin (foreland basin; Larsen, 1988; Petersen *et al*., 2016). In Billefjorden, Eurekan deformation involved the NE-dipping Cowantoppen Fault, which accommodated up to 200 m of top-SW reverse movement, and the southern continuation of the Balliolbreen Fault, the east-dipping Gipshuken Fault, which offsets the Wordikammen and Gipshuken formations by up to 200 m of top-west ( Bælum & Braathen, 2012; Dallmann *et al*., 2004a; Harland *et al*., 1974; Ringset & Andresen, 1988).

## Methods

**Figure 3.**
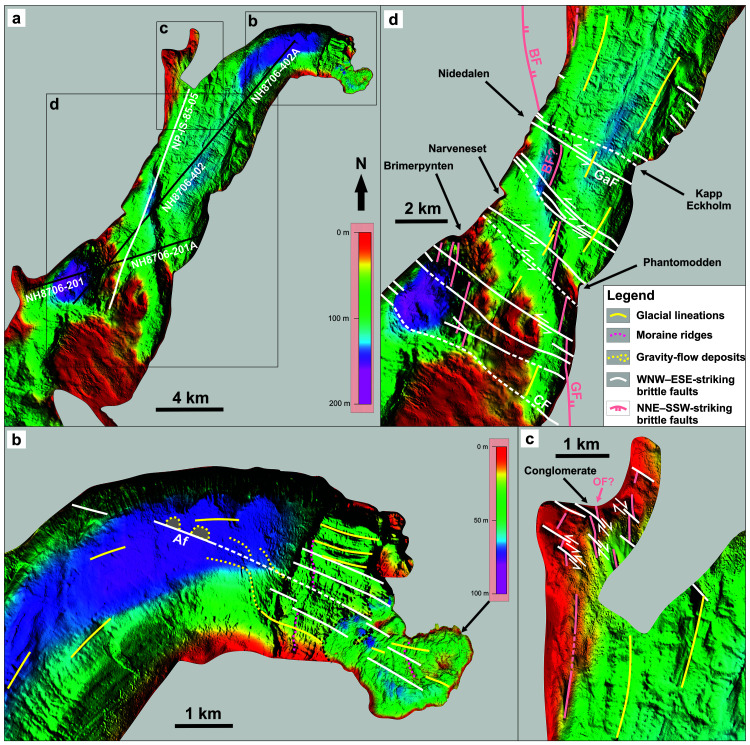
( **A**) Bathymetric data in Billefjorden (see Figure 1A for location) showing the location of seismic line NP-IS-85-05 ( Figure 4A–B) as a white line and of other seismic lines included as supplements as black lines. ( **B**– **D**) Zooms in ( **B**) Adolfbukta and Nordenskiöldbreen, ( **C**) Pyramiden, and ( **D**) southern Billefjorden showing dominant WNW–ESE-trending, fault-related escarpments (white lines; including the Adolfbukta fault) offsetting (dominantly left-) laterally subsidiary NNW–SSE- to NNE–SSW-trending fault-related escarpments and ridges that parallel known major faults (e.g., Balliolbreen and Odellfjellet faults). Both sets of escarpments trend oblique to local glacial lineations (yellow lines). The reader is referred to Baeten *et al.* (2010) and Allaart *et al.* (2018) for detailed interpretation of glacial features in the area. Note the hundreds of meter- to kilometer-scale left-lateral offsets of a NNW–SSE-trending ridge consisting of NNE–SSW-striking, Z-shaped (possibly drag-folded) segments of the Balliolbreen Fault by WNW–ESE-striking faults in ( **D**). Grey shading denotes areas with no data. The dotted black line in ( **B**) indicates the boundary between the two bathymetric datasets with different color-scale. Abbreviations: Af – Adolfbukta fault; BF – Balliolbreen Fault; CF – Cowantoppen Fault; GaF – Garmaksla fault; GF – Gipshuken Fault; OF – Odellfjellet Fault. This figure was designed and copyright is held by the authors of the present manuscript.

**Figure 4.**
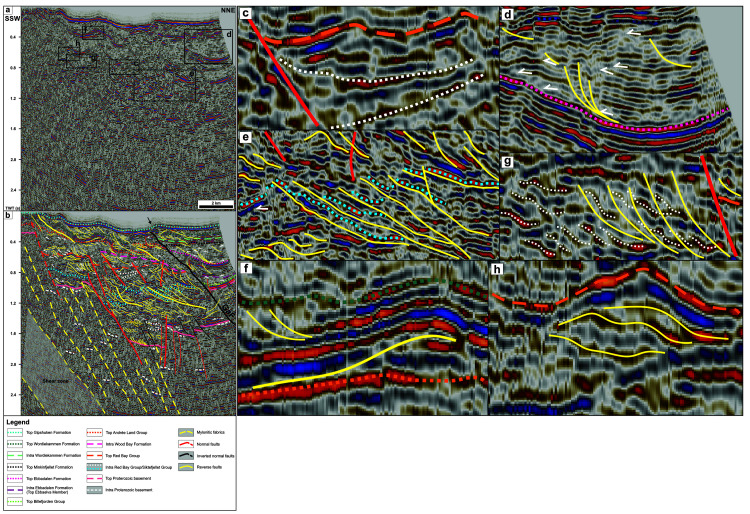
( **A**) Uninterpreted and ( **B**) interpreted seismic line NP-IS-85-05 in Billefjorden showing dominant WNW–ESE-trending, NNE-dipping ductile fabrics in Proterozoic basement rocks, and Early Devonian to Pennsylvanian (mildly inverted) normal faults and associated early Cenozoic overprints in Lower Devonian to lower Permian sedimentary rocks. See Figure 1B for location. ( **C**– **H**) Zooms in ( **C**) a wedge of Lower Devonian sedimentary rocks (dotted white lines) thickening against a NNE-dipping, high-angle, Devonian normal fault (red line) that was partially inverted in early Cenozoic times as shown by gently folded seismic reflections (e.g., dashed orange line), ( **D**) an imbricate thrust fan (yellow lines) and onlaps and toplaps (white half-arrows) within the Minkinfjellet Formation, ( **E**) early Cenozoic low-angle thrusts (yellow lines) and thrust sheets (dotted blue lines) within Lower Devonian sedimentary rocks, ( **F**) a top-NNE early Cenozoic Eurekan thrust (yellow line) flattening and soling into the stratigraphic boundary between the Wood Bay Formation and Wordiekammen Formation (dotted orange line), thus suggesting the presence of a décollement, ( **G**) folded bedding surfaces (dotted white lines) offset by top-SSW thrust faults (yellow lines) and arranged into duplexes within Lower Devonian sedimentary rocks, and ( **H**) an antiformal thrust stack or ramp anticline within Lower Devonian sedimentary rocks. Note that the bedding surfaces arranged in top-SSW duplexes shown in ( **G**) are not displayed in ( **B**) due to insuffiencient resolution. Abbreviations: GaF – Garmaksla fault. The seismic data in the figure is reproduced with permission from copyright holders, Norwegian Petroleum Directorate.

The study presents structural analysis of submarine escarpments on bathymetric data from the Norwegian Mapping Authority and the University Centre in Svalbard ( Figure 3A–D and electronic supplement 2) and on Two-Way Time (TWT) seismic data from the Norwegian National Data Repository for Petroleum Data (DISKOS database) in Billefjorden ( Figure 4A–H and electronic supplements 3 and 4). See *Underlying data* for full details of the datasets used. To interpret bathymetric and seismic data, we used Global Mapper (version 13) and Petrel (version 2021.3) respectively. QGIS and OpendTect are free, open source alternative software that can be used to perform similar functions to Global Mapper and Petrel respectively. CorelDraw 2017 ( GIMP is a freely available open source alternative) was used to design the figures.

The depth and thickness of sedimentary units were obtained through interpretation of the main sedimentary unit boundaries and brittle faults and shear zones on seismic data ( Figure 4A–H and electronic supplements 3 and 4). The interpretation of the various stratigraphic units is included in electronic supplement 5. Velocity data are taken from exploration well 7816/12-1 in Reindalspasset (electronic supplement 6; Eide *et al*., 1991) and from Gernigon *et al*. ( 2018, their Table 1), and were used to calculate estimates (minimum and maximum) of depths and thicknesses (in meters – m) from Two-Way Time seismic data. We used velocities from nine intervals (1850 to 2250 m; depths of intervals are specified *Extended data*) in well 7816/12-1 to calculate an average velocity for Pennsylvanian rocks in central Spitsbergen (c. 5940 m.s ^-1^) and used average velocities from Gernigon *et al*. (2018) of 5500 m.s ^-1^ for Pennsylvanian and 5500 to 5800 m.s ^-1^ for Devonian to Mississippian sedimentary rocks. For the depth conversion of the seismic section (electronic supplement 7), we used the velocities from Gernigon *et al*. ( 2018; electronic supplement 6). High-resolution versions of the manuscript’s figures (necessary to identify the structures and stratigraphic units mentioned) can be found in *Underlying data * ( Koehl *et al*., 2023c) and *Extended data (* Koehl *et al*., 2023d).

## Results and interpretations

### Bathymetric data

***Description***

In Adolfbukta, bathymetric data ( Figure 3A; see high-resolution versions of the figure at *Underlying data*) the mouth of Nordenskiöldbreen show numerous narrow (several meters wide), undulating to arcuate, NNE–SSW- to N–S-trending, ice-margin parallel, typically 2 to 3 m high submarine ridges that were interpreted as moraines deposited during the most recent recession of Nordenskiöldbreen (dotted fuchsia lines in Figure 3B; Allaart *et al*., 2018). Farther south, the moraine ridges appear to abruptly bend into a NNW–SSE trend across localized WNW–ESE-trending submarine escarpments that accommodate gentle to moderate (typically 5 to tens of meters) drops in bathymetry ( Figure 3B and electronic supplement 8). The WNW–ESE-trending escarpments align with but are distinct from widespread, smooth, WNW–ESE-trending, oval-shaped hills and parallel lineations and troughs, which were interpreted as glacially streamlined landforms (drumlins and glacial lineations) and mass transport deposits with associated channels (yellow lines in Figure 3B; Allaart *et al*., 2018; Baeten *et al*., 2010). Glacial lineations in the deepest portion of the fjord show arcuate geometries, bending from an E–W trend in the northeast to a NE–SW trend in the southwest, i.e., following the fjords attitudes. Oblique to the glacial lienations is a subtle WNW–ESE-trending lineament bounding two 200–300 m wide, 4–5 m high, WNW–ESE-trending, lens-shaped ridges (dotted yellow lines; Figure 3B), which coincide with the end of gravity flow tracks ( Allaart *et al*., 2018).

 In the northwestern part of Billefjorden near Pyramiden, bathymetric data show a few discontinuous, steep, N–S- and WNW–ESE-striking submarine escarpments abutting and/or crosscutting one another ( Figure 3C). Notably, one of the main N–S-trending escarpment, which defines the western flank of a c. 1 km-long ridge, appears offset right-laterally by c. 100 m by a series of aligned, discontinuous, WNW–ESE- to NW–SE-trending lineaments and escarpments ( Figure 3C).

 In the southern part of Billefjorden, bathymetric data reveal a kilometer-wide, overall N–S-trending ridge that is bounded to the west by alternating steep, N–S- to NNE–SSW-trending escarpments and relatively smooth, arcuate (anticlockwise-bending), NNW–SSE-trending escarpments that define hundreds of meter- to kilometer-scale (500 to 2000 m) left steps along the ridge axis ( Figure 3D). The smoother and arcuate NNW–SSE-trending portions of this ridge line up with steep, subvertical, high-frequency, WNW–ESE- to NW–SE-trending escarpments on the edges of the fjord along a WNW–ESE- to NW–SE-trending axis on bathymetric data ( Figure 3D).

***Interpretation***

The location of the N–S-trending escarpment near Pyramiden coincides with the suspected continuation of the Odellfjellet Fault ( Braathen *et al*., 2011; Smyrak-Sikora *et al*., 2018) and with that of a Eurekan thrust ( Dallmann *et al*., 2004a) and is therefore interpreted as a brittle fault. In southern Billefjorden, the kilometer-wide, overall N–S-trending ridge and associated escarpments trend sub-orthogonal to and display significantly different geometries from smooth, undulating, WNW–ESE- to E–W-trending narrow ridges interpreted as recessional moraines ( Baeten *et al*., 2010). The ridge aligns with the speculated location and strike of the Balliolbreen Fault in Lykteneset ( Braathen *et al*., 2011; Dallmann *et al*., 2004a; Harland *et al*., 1974). The ridge may therefore represent the nearshore southern continuation of the Balliolbreen Fault. However, the ridge also coincides with landslides involving rocks of the Wordiekammen Formation onshore in the hanging wall of the Balliolbreen Fault ( Dallmann *et al*., 2004a; Koehl *et al*., 2022 submitted, their supplement S8). Hence, the ridge may reflect east-dipping carbonate beds of the Wordiekammen Formation that are offset by the Balliolbreen Fault and/or are part of the western limb of the Petuniabukta Syncline ( Braathen *et al*., 2011; Maher & Braathen, 2011). The latter is supported by the smooth and gently east-dipping character of the top of the ridge and by the steeply dipping western flank of the ridge on bathymetric profiles (electronic supplement 9), and by the gently dipping attitude of sedimentary strata within the ridge on seismic data (see black arrow in Figure 4B).

WNW–ESE-trending escarpments in Billefjorden are interpreted as brittle faults. This is supported by the following arguments. First, WNW–ESE-trending escarpments parallel and, in places, align with onshore fault-related escarpments observed on satellite images and in the field ( Figure 3B–D, electronic supplement 10, Koehl *et al*., 2023a their figure 3a–b, and Koehl *et al*., 2023b their figures 2, 3, 4a and d–e, and 5). Second, the geometry of the WNW–ESE-trending escarpments (steep and discontinuous) contrasts with that of smooth and continuous, WNW–ESE-trending ridges interpreted as recessional moraines ( Baeten *et al*., 2010; Figure 3B–C). This is also the case for N–S-trending fault-related escarpments and NNE–SSW-trending lineations previously interpreted as glacial lineations in northwestern Billefjorden and ( Baeten *et al*., 2010; Figure 3C). Third, the curving, abutting and crosscutting relationships of N–S- and WNW–ESE-striking escarpments suggest lateral tectonic offsets and/or stepping attitudes of brittle faults. Notably, in southern Billefjorden, the left-stepping and anticlockwise-bending geometry of the smoother and arcuate NNW–SSE-trending portions of the overall N–S-trending ridge (Balliolbreen Fault?) are interpreted to reflect 0.5 to 1.5 km wide offsets and drag-folding by NW–SE- to WNW–ESE-striking faults. Fourth, in places (e.g., northeastern Billefjorden), WNW–ESE-trending escarpments bound mass-flow deposits and are highly oblique to local glacial features ( Figure 3B; Allaart *et al*., 2018).

The smoother, arcuate, NNW–SSE-striking portions of the Balliolbreen Fault link up segments characterized by well defined, steep, N–S- to NNE–SSW-trending escarpments ( Figure 3D). Based on the numerous occurrences and, in places, dominance of N–S- to NNE–SSW-striking faults in Billeforden ( Figure 3C; Koehl *et al*., 2023a their fig. 3A–B, 5a, c, and d, and 6b; Koehl *et al*., 2023b, fig. 2–6), we propose that NNE–SSW- to N–S-trending fault-related escarpments along the Balliolbreen Fault represent the actual strike of the fault, whereas relatively smoother and arcuate, NNW–SSE-striking segments represent portions of the fault that were drag-folded and offset by (c. 0.5 to 1.5 km-wide) left-lateral movements along WNW–ESE-striking faults ( Figure 4A–B).

### Seismic data

***Structures in Proterozoic basement rocks***

Description

 Highly reworked basement rocks commonly display chaotic facies in seismic data (e.g., Ji & Long, 2006; Koehl *et al*., 2018; Phillips & McCaffrey, 2019). However, the deepest portions of seismic sections in Billefjorden show sub-horizontal to gently NNE-dipping, undulating, low to moderate-amplitude reflections (dashed white lines in Figure 4B). These are extensively disrupted and truncated by moderately to steeply NNE-dipping (gently dipping once depth-converted; electronic supplement 7), sub-planar and sub-parallel, moderate-amplitude reflections (dashed yellow lines in Figure 4B), which dominate in the south-southwest at a depth > 1.2 second TWT, i.e., > 7 km (grey-shaded area in Figure 4B and electronic supplement 7; see high-resolution versions of the figure in *Underlying data*). Upwards, these sub-horizontal to gently NNE-dipping low to moderate-amplitude reflections are truncated at an angle by a mildly undulating, moderate to high-amplitude, negative reflection interpreted as the Top Proterozoic basement reflection.

Interpretation

The sub-horizontal undulating reflections are interpreted as lithological transitions within folded Proterozoic basement rocks. These attitudes are similar to intra-unit lithological transitions in folded metasedimentary rocks and metaigneous rocks crosscut by intrusions in nearby onshore areas ( Bayly, 1957; Christophersen, 2015; Dallmann *et al*., 2004a; Koehl *et al*., 2023b), and to folded basement rocks on seismic data worldwide and in Svalbard ( Ji & Long, 2006; Koehl & Allaart, 2021).

The disruptive and truncating character of moderately to steeply (gently when depth-converted; electronic supplement 7) NNE-dipping, moderate-amplitude reflections suggests that they represent pervasive WNW–ESE-trending, NNE-dipping, brittle to ductile, possibly mylonitic fabrics within Proterozoic basement rocks. This is supported by the dominance of WNW–ESE-trending brittle to ductile fabrics in Proterozoic basement rocks in adjacent onshore areas and on bathymetric data in Billefjorden (e.g., Figure 3B and Koehl *et al*., 2023b their figures 2–6), and by the strong influence of preferred mineral orientation on seismic velocity ( Christensen, 1965; Fountain *et al*., 1984; Hurich *et al*., 1985). The high concentration of potential mylonitic surfaces within a kilometer-thick zone in the south-southwest (gray-shaded area in Figure 4A–B) indicates that these deformation surfaces are probably aggregated into a major NNE-dipping shear zone, which displays a similar seismic facies to other mylonitic shear zones worldwide (e.g., Christensen & Szymanski, 1988; Clerc *et al*., 2018; Fazlikhani *et al*., 2017; Fisher *et al*., 1989; Hajnal *et al*., 1996; Hedin *et al*., 2016; Koehl *et al*., 2018; Lenhart *et al*., 2019; Osagiede *et al*., 2020; Phillips & McCaffrey, 2019; Phillips *et al*., 2016; Wang *et al*., 1989; Wrona *et al*., 2020).

***Structures in post-Caledonian sedimentary rocks***

Devonian to Carboniferous extensional normal faults and folds

Description

Stratigraphic units and sub-units and internal reflections within Devonian to Carboniferous sedimentary rocks are offset by several high-angle (moderately dipping when depth converted; electronic supplement 7), sub-planar disruption surfaces that bound north-northeastward-thinning wedge- to fan-shaped seismic sub-units interpreted as alluvial fans (see also electronic supplement 5). Most of these disruption surfaces dip north-northeastwards, terminate upwards within the Wood Bay Formation or the Hultberget and/or Ebbadalen formations, and coincide with abrupt northwards thickening of overlying and adjacent wedge- to fan-shaped stratigraphic units and sub-units. In places, the disruption surfaces are subvertical, dip to the south-southwest, terminate in the lower part of the Devonian succession, and offset seismic reflections interpreted as stratigraphic boundaries and bedding surfaces down-SSW ( Figure 4A–B). Both NNE- and SSW-dipping disruption surfaces crosscut stratigraphic (sub-) unit boundaries and bedding surfaces (e.g., dotted blue lines) at a high-angle ( Figure 4A–C). Downwards, NNE-dipping disruptions surfaces dip slightly more gently (subtle listric geometry) and merge with parallel mylonitic fabrics in the hanging wall of the kilometer-thick shear zone in underlying and adjacent Proterozoic basement rocks ( Figure 4A–B).

In the north-northeast near Pyramiden, Pennsylvanian strata of the Hultberget and/or Ebbadalen and Minkinfjellet formations are involved in a 4 to 5 km-wide, open, E–W- to WNW–ESE-trending syncline ( Figure 4A–B & D). There, strata of the Minkinfjellet Formation are thickest near the center of the syncline and terminate as toplaps on the edge of the syncline against a flat-lying Top Minkinfjellet Formation reflection.

Interpretation

Based on their listric geometries, on their high-angle crosscutting relationships with bedding surfaces and stratigraphic (sub-) unit boundaries, on the normal offsets of stratigraphic (sub-) units boundaries across these surfaces, on abrupt thickening of stratigraphic units and sub-units over these surfaces (i.e., possibly representing syn-tectonic deposits), and on their absence in Middle Pennsylvanian to lower Permian strata of the Minkinfjellet, Wordiekammen and Gipshuken formations, we interpret the high-angle (modetarely dipping when depth converted; electronic supplement 7) disruption surfaces as Early Devonian to Early Pennsylvanian normal faults. Normal offsets along these faults are in the range of hundreds of meters to 2 km. For example, the second southernmost of these faults displays the largest offset and potentially offsets the Top Basement reflection by c. 0.7 second (TWT), i.e., ca. 1.9 to 2.0 km, and reflections within the Siktefjellet and/or Red Bay groups (e.g., dotted blue line reflection) by up to ca. 0.3 second (TWT), i.e., c. 825 to 870 m (electronic supplement 6) down-NNE ( Figure 4A–B). These brittle, dominantly NNE-dipping normal faults show similar parallel and merging relationships to preexisting basement fabrics as normal faults in Devonian to Carboniferous collapse basins in the Barents Sea ( Koehl *et al*., 2018; Koehl *et al*., 2022a) and North Sea ( Fazlikhani *et al*., 2017; Lenhart *et al*., 2019; Osagiede *et al*., 2020; Phillips *et al*., 2016). They are therefore interpreted to reflect late- to post-Caledonian Devonian (to Early Pennsylvanian?) collapse along partly reactivated, inherited, NNE-dipping basement fabrics.

The synclinal fold structure most likely partly reflects the contrasting characters of syn-tectonic Lower Pennsylvanian rocks and latest to post tectonic sedimentary strata of the Minkinfjellet Formation. The former were deposited as a potential alluvial fan along a NNE-dipping normal fault, which dies out in the middle to upper part of the Lower Pennsylvanian succession, whereas the latter passively filled accommodation space created by down-NNE normal faulting. This is supported by northward thickening of the Minkinfjellet Formation and northwards thinning of Lower Pennsylvanian strata of the Hultberget and/or Ebbadalen formations on the southern limb, and by southward thickening of the Minkinfjellet Formation and southwards tilting (and northward thinning) of Lower Pennsylvanian strata against a NNE-dipping normal fault on the northern limb ( Figure 4A–B).

Early Cenozoic thrusts and contractional duplexes

Description

Interpreted stratigraphic units and sub-units and intra-unit reflections are truncated and offset by numerous low-angle disruption surfaces showing diverse geometries including low-angle and sub-planar, listric and fan-shaped, Z-like, and upwards-convex geometries. Low-angle and sub-planar, dominantly NNE-dipping disruption surfaces commonly show top-SSW reverse offsets and, occasionally, (S-like) bending of stratigraphic (sub-) units boundaries (e.g., Top Red Bay Group reflection) and bedding surfaces (e.g., dotted blue lines in Figure 4B & E). In places, these disruption surfaces merge with bedding surfaces (e.g., dotted blue lines) but die out rapidly laterally, extending less than 3 km from north-northeast to south-southwest ( Figure 4A–B & E).

Z-like reflections extending laterally for tens to hundreds of meters are particularly abundant within Devonian (Siktefjellet and/or Red Bay groups and Wood Bay Formation) and lower Permian (Wordiekammen and Gipshuken formations) units. These reflections mostly occur as groups of several adjacent reflections bounded upwards and downwards by planar reflections ( Figure 4A–B & G).

Interpretation

The low-angle disruption surfaces are interpreted as early Cenozoic thrust faults locally bounding and detaching thin (<< 0.1 s TWT thick) thrust sheets and soling into bedding-parallel décollements ( Figure 4A–B & E–F). This is suggested by the low-angle relationship with bedding surfaces and stratigraphic (sub-) unit boundaries, the reverse offsets of stratigraphic boundaries and related seismic reflections, the narrow (< 3 km) lateral extent of thrust faults, and their presence in all stratigraphic units. The only exception to the limited lateral extent is a thrust detaching a c. 1 km-wide, open, SSW-verging anticline fold structure in the hanging wall involving a ca. 0.2 s (TWT) thick succession of strata of the Wood Bay, Wordiekammen and Gipshuken formations. In the north-northeast, this thrust fault merges with the Top Andrée Land Group (top of Lower Devonian) stratigraphic boundary that potentially hosts a local, bedding-parallel décollement detaching the overlying SSW-verging anticline ( Figure 4A–B & F). This suggests that this fold structure corresponds to a ramp anticline. In places, low-angle thrusts display listric and downwards merging geometries, forming fan-shaped structures that may represent imbricate thrusts ( Figure 4A–B & D). Offsets along low-angle thrusts are in the range of (tens to hundreds of) meters and may reach up to 1 to 2 km in places (e.g., offset dotted blue line in Figure 4B & E).

Z-like reflections may well correspond to tilted prograding sedimentary systems. However, since the rounded edges of these Z-like reflections seems to coincide with subtle bends in bounding planar reflections ( Figure 4A–B & G), and considering their geometrical similarity with low-angle thrusts and their occurrence as packages of adjacent Z-shaped reflections, we propose that they correspond to brocken-up, stacked bedding surfaces in contractional duplexes ( Boyer & Elliott, 1982) most likely consisting of roof- and floor-thrusts ( McClay, 1992) connected by link thrusts ( McClay & Insley, 1986). The seismic expression and geometry of these structures is very similar to contractional duplex structures in uppermost Devonian to Mississippian coal-rich sedimentary deposits of the Billefjorden Group onshore in Pyramiden and nearshore in Sassenfjorden and Tempelfjorden ( Koehl, 2021). Aggregates of contractional duplexes commonly align with the imaginary downwards and upwards prolongation of low-angle subplanar thrusts, seemingly connecting them and, thus, possibly acting as hard- to soft-linking transfer structures ( Figure 4A–B). In places, Z-shaped reflections show upwards-convex geometries ( Figure 4A–B & H), which may also represent contractional duplexes, but that potentially suggest tilting and reworking of the duplexes into local antiformal thrust stacks.

Contractional inversion structures

Description

Devonian to Permian strata in Billefjorden are also deformed into multiple kilometer-scale fold structures. Notably, Devonian and lower Permian strata in the south are involved into a 1 to 2 km-wide open anticline, which location coincides with that of the tip of high-angle normal faults terminating within the Wood Bay Formation ( Figure 4A–B).

In the north-northeast, a major, moderately NNE-dipping, gently undulating disruption surface abuts near the seafloor reflection and merges at depth with the main NNE-dipping fault bounding uppermost Devonian to Pennsylvanian sedimentary strata ( Figure 4A–B). This disruption surface coincides with a break and a minor (c. 500 m-wide) bump in the seafloor reflection, and with a top-SSW, ca. 0.1 second (TWT) (i.e., c. 250 to 325 m; electronic supplement 6) reverse offset of the Top Wordiekammen reflection ( Figure 4A–B and electronic supplement 5).

Interpretation

It is possible that the 1 to 2 km-wide open anticline is related to upwards propagation of normal faults in post-Carboniferous times. However, considering its occurrence at the same depth and its proximity to the interpreted ramp anticline in the hanging wall of an early Cenozoic low-angle thrust 1 to 2 km farther south, it is more probable that this broad anticline reflects mild inversion of preexisting Devonian normal faults during early Cenozoic contraction.

Considering the merging relationship with the high-angle normal fault at depth, reverse offset of the Top Wordiekammen Formation, and minor break and bump in the seafloor reflection ( Figure 4A–B), we argue that the major, moderately NNE-dipping, gently undulating disruption surface represents an early Cenozoic top-SSW thrust overprint of a major (Devonian? to) Early Pennsylvanian down-NNE normal fault. Based on the absence of Pennsylvanian sedimentary strata of the Hultberget, Ebbadalen and Minkinfjellet formations south of this fault on seismic data and on a subtle (c. 250 to 325 m, i.e., ca. 0.1 s TWT; electronic supplement 6) thickness increase of the Wordiekammen Formation in the fault hanging wall, the fault is thought to have initiated as a Pennsylvanian to earliest Permian normal fault, accommodating as much as 1.1 to 1.2 km of down-NNE normal displacement (i.e., ca. 0.4 s TWT; electronic supplement 6) and was later reactivated as a thrust in the early Cenozoic. This fault is associated with a 4 to 5 km-wide, open, E–W- to WNW–ESE-trending syncline in rocks of the Billefjorden and Gipsdalen groups in the hanging wall ( Figure 4A–B & D). Though this fold is partly thought to reflect the late- to post-tectonic character of Middle Pennsylvanian deposits of the Minkinfjellet Formation, involvement of the Top Basement and Top Billefjorden Group reflections in the folding ( Figure 4A–B) suggest that it (at least partly) formed due to Eurekan contraction in the early Cenozoic.

### Correlation of seismic and bathymetric data and satellite images

The locations of dominantly NNE-dipping brittle faults on seismic data ( Figure 4A–B) correlate well with WNW–ESE-striking fault-related escarpments on bathymetric data ( Figure 3D). Notably, (1) the main NNE-dipping, inverted normal fault bounding uppermost Devonian to Pennsylvanian strata on seismic data in Billefjorden and presently named the Garmaksla fault (250 to 325 m top-SSW offset; electronic supplement 6), (2) the system of hard- to soft-linked early Cenozoic, Eurekan, top-SSW thrusts (250 to 325 m top-SSW offset; electronic supplement 6), and (3) the major low-angle, early Cenozoic, NNE-dipping partial décollement (c. 1 km top-SSW offset; Figure 4A–B) coincide with WNW–ESE-striking fault-related escarpments with associated drag folding and left-lateral offsets of the Balliolbreen Fault that extend respectively (1) from a few hundreds of meters north of Nidedalen to Kapp Eckholm (0.5 to 1.5 km offset), (2) from Nidedalen to c. 1 km northeast of Phantomodden (offset up to 1.5 km), and (3) from southwest of Brimerpynten to southwest of Phantomodden (offset of up to c. 500 m; Figure 3D). The drag-folding in map view and both horizontal ( Figure 3D) and vertical offsets across these faults ( Figure 4A–B) suggest that they accommodated oblique-slip sinistral-reverse movements in the early Cenozoic.

Alternatively, the apparent left-lateral offset of the ridge on bathymetric data ( Figure 3D) may reflect vertical offset of gently dipping sedimentary strata of the Wordiekammen Formation, as suggested from bathymetric profiles (electronic supplement 9) and onshore studies in Garmdalen and Lykteneset ( Dallmann *et al*., 2004a; Koehl *et al*., 2022 submitted). If they were purely vertical, the offsets along WNW–ESE-striking faults must therefore be normal down-NNE or reverse top-NNE because of the east-dipping character of sedimentary strata. Seismic data show that WNW–ESE-striking faults offsetting the ridge dip to the north-northeast and accommodated top-SSW offset of the Wordiekammen Formation (e.g., Garmaksla fault; Figure 4A–B), therefore suggesting that the WNW–ESE-striking faults in Billefjorden did accommodate some sinistral strike-slip movement component. This is further supported by slickenside lineations indicating sinistral to sinistral-reverse movement along WNW–ESE-striking faults in Narveneset (electronic supplement 11).

Considering the NNE–SSW strike of the Balliolbreen Fault ( Figure 3A & D), i.e., parallel to the NNE–SSW-trending seismic line in Figure 4A–B, it is very likely that the fault should appear as a horizontal reflection or alignment of reflection disruptions, at least in the northern half of the seismic line since the laterally offset northern segment of the Balliolbreen Fault dips ESE and, thus, most likely intersects with the seismic section at depth. Taking into account previous studies of the Balliolbreen Fault in the area (e.g., Harland *et al*., 1974; Koehl, 2021), it is probable that the Balliolbreen Fault actually localized within weak, coal-rich sedimentary deposits of the Billefjorden Group (see dashed red line in Figure 4B) as observed in Pyramiden ( Koehl, 2021), or simply separates the Billefjorden Group from Proterozoic basement rocks (i.e., dashed light red line in Figure 4B).

## Discussion

The discussion (1) reviews evidence for the presence of a fault in northern Billefjorden, the Adolfbukta fault and (2) evaluates the impact of newly identified WNW–ESE-striking faults on the Billefjorden Trough and related faults. Then, the discussion addresses the implications of the present study for (3) post-Caledonian extension at a regional scale (a detailed discussion of further implications for Devonian–Carboniferous normal faulting is included as electronic supplement 12), (4) the Svalbardian Orogeny and the Eurekan tectonic event, and (5) the Petuniabukta Syncline. Finally, a brief account on (6) the orogin of the WNW–ESE-striking faults in Billefjorden is given.

### The Adolfbukta fault

In Pyramiden, relatively recent studies (e.g., Bergh *et al*., 2011; Michaelsen *et al*., 1997; Piepjohn *et al*., 1997; Piepjohn, 2000) suggested the presence of Proterozoic basement rocks in outcrops below the entrance of the Russian coal mine of Pyramiden, although older works showed only Devonian to Mississippian sedimentary rocks ( Harland *et al*., 1974; Lamar *et al*., 1986; Sirotkin, pers. comm. 2019). Recent fieldwork and thin section analysis on both sides of the speculated trace of the Balliolbreen Fault in this area further supports the absence of basement in Pyramiden ( Koehl, 2021). The contact between Proterozoic basement and uppermost Devonian to Permian sedimentary rocks, which crop out at a maximum altitude of c. 500 m in Elsabreen 2 to 2.5 km north of Pyramiden ( Figure 1B), is therefore located below ground level in Pyramiden, i.e., at least below an altitude of 100 m. Similarly in eastern Billefjorden, the unconformity between Proterozoic basement rocks and overlying uppermost Devonian to Mississippian rocks of the Billefjorden Group shows a comparable southwards altitude decrease across Adolfbukta, from > 200 m altitude in De Geerfjellet ( Dallmann *et al*., 2004a; svalbardkartet.npolar.no) to a depth of 116 m in Brucebyen ( Cutbill *et al*., 1976).

In addition, several stratigraphic units thicken southwards across Adolfbukta. For example, the thickness of the Billefjorden Group increases from < 40 m in De Geerfjellet (see location in Figure 1B) to 85 to 100 m-thick in Brucebyen ( Figure 1B; Cutbill *et al*., 1976). The uppermost Devonian to Middle Pennsylvanian sedimentary infill (Billefjorden Group and Hultberget, Ebbadalen, and Minkinfjellet formations) in Petuniabukta is c. 1.3 km thick based on exploration wells of Trust Arktikugol ( Senger *et al*., 2019). These successions thicken to the south to c. 1.65 to 1.78 km based on depth conversion of seismic data near Pyramiden (ca. 0.6 s TWT; Figure 4A–B; electronic supplement 6). Such significant and abrupt thickening to the south may reflect c. 350 to 480 m of syn-sedimentary, down-SSW normal movements along an E–W- to WNW–ESE-striking fault zone in Adolfbukta, including up to 45 to 60 m during the deposition of the Billefjorden Group based on thickness variations ( Cutbill *et al*., 1976). A potential candidate is the Kampesteindalen Fault ( Smyrak-Sikora *et al*., 2018). However, the estimated total normal displacement along the Kampensteindalen Fault is thought to be around 50 m in the Early Pennsylvanian ( Smyrak-Sikora *et al*., 2018), i.e., much smaller than total southward sedimentary thickness increase across Adolfbukta. Displacement along the Kampesteindalen Fault is also much smaller than the 300 to 400 m southward altitude drop of the contact between Proterozoic basement rocks and the Billefjorden Group. It is therefore unlikely that the Kampesteindalen Fault is, alone, responsible for such southwards deepening and thickening. This therefore suggests the presence of a larger SSW-dipping normal fault extending between Adolfbukta and Pyramiden, the Adolfbukta fault. This is further supported by numerous WNW–ESE-striking fault-related escarpments in Proterozoic basement rocks on satellite images, in the field ( Koehl *et al*., 2023b, figure 2, 3, 4a, d–e, and 5) and on bathymetric data in Nordenskiöldbreen ( Figure 3A–D), and in nearby onshore areas in Billefjorden ( Witt-Nilsson *et al*., 1998, figure 3; Christophersen, 2015; Koehl & Muñoz-Barrera, 2018; Koehl *et al*., 2023a, figure 3A–B; Koehl *et al*., 2023b, figure 2, 3, 4a, d–e, and 5). It is also supported by similarly striking normal faults on seismic data in Billefjorden, which show comparable, several hundreds of meter- to kilometer-scale normal offsets of uppermost Devonian to Permian sedimentary successions ( Figure 4A–B).

The inferred SSW-dipping Adolfbukta fault defines a WNW–ESE-trending lineament that is highly oblique to glacial features and bounds two, several meters high, WNW–ESE-trending lensoidal ridges in the deepest part of the fjord in Adolfbukta (dotted yellow lines in Figure 3B). In the west, the Adolfbukta fault may continue in Hugindalen where Piepjohn *et al*. ( 1997, figure 4) and Piepjohn ( 2000, figure 3) mapped a south- to SSW-dipping brittle normal fault that aligns with the inferred trace of the Adolfbukta fault in Adolfbukta and between Elsabreen and Pyramiden ( Figure 1B). East of Billefjorden, the Adolfbukta fault may extend as far as Rembebreen based on mapping by the Norwegian Polar Institute (svalbardkartet.npolar.no), which shows a WNW–ESE-striking fault in the area.

Alternatively or complementarily to the Adolfbukta fault, the 300 to 400 m altitude drop of the top of the Proterozoic basement and base of the Billefjorden Group from Elsabreen to Pyramiden and from De Geerfjellet to Brucebyen may be related to the presence of a several kilometers wide, WNW–ESE-trending, SSW-verging fold structure. Seismic data document the presence of multiple WNW–ESE-trending, open, upright fold structures, including a 4 to 5 km-wide, open, E–W- to WNW–ESE-trending syncline within rocks of the Billefjorden and Gipsdalen groups in northern Billefjorden ( Figure 4A–B & D), which are most likely (at least partly) related to mild early Cenozoic inversion of Early Devonian to Carboniferous normal faults during Eurekan deformation. In addition, recent field studies reveal that the N–S-trending, west-verging Mimerelva Syncline ( Piepjohn, 2000) bends into a WNW–ESE-trending, SSW-verging geometry in Munindalen ( Koehl & Stokmo, 2021a; Koehl & Stokmo, 2021b; Koehl *et al*., in prep.), i.e., in the west-northwestwards prolongation of the inferred SSW-verging fold between Elsabreen and Pyramiden. E–W- to WNW–ESE-trending early Cenozoic fold structures in Billefjorden would also explain the enigmatic attitude of gently NNE-dipping bedding surfaces and stratigraphic boundaries within the Lower Devonian Wood Bay Formation and uppermost Devonian–Mississippian Billefjorden Group above the entrance of the coal mine in Pyramiden ( Koehl, 2021). Nonetheless, the presence of numerous WNW–ESE-trending fault-related escarpments on bathymetric ( Figure 3B) and in the field in Adolfbukta ( Koehl *et al*., 2023b, figures 4a and d–e, and 5b) and the horst-and-graben geometries defined by WNW–ESE-striking faults at Top Proterozoic basement level on seismic data ( Figure 4A–B) suggest that initial basin geometry bounded by WNW–ESE-striking normal faults played a role in the observed variations. Therefore, the more likely scenario is an interplay between a Devonian to Carboniferous (SSW-dipping) normal fault and a related early Cenozoic, SSW-verging fold structure (e.g., electronic supplement 13).

### Segmentation of the Billefjorden Trough by WNW–ESE-striking faults

**Figure 5.**
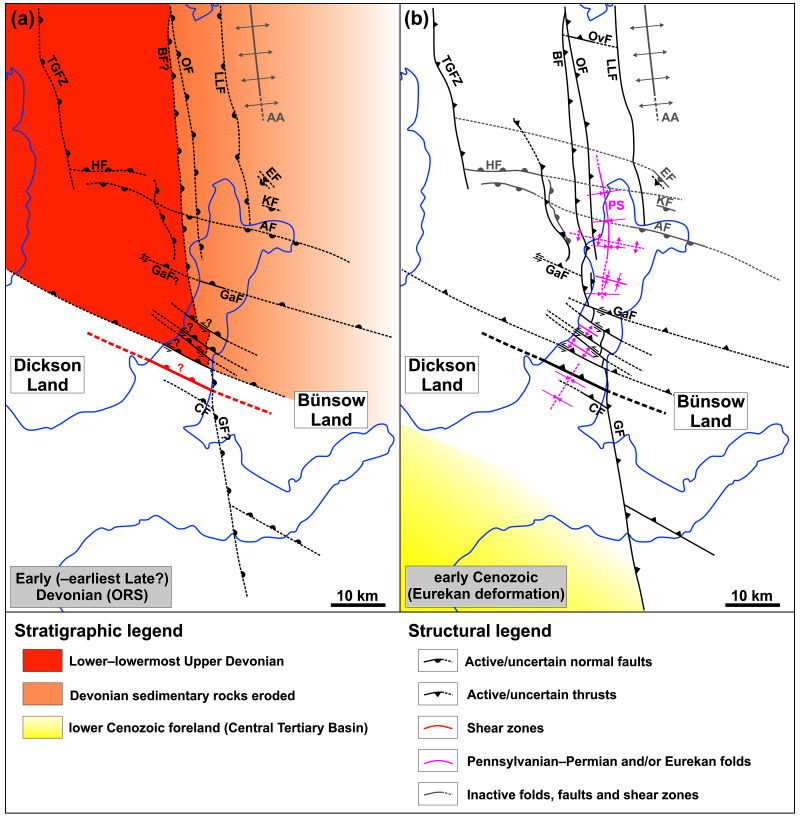
Post-Caledonian tectonic history of the Billefjorden and Sassenfjorden areas including ( **A**) (late Silurian? to) Devonian extensional collapse along major NNE-dipping normal faults reactivating/overprinting WNW–ESE-striking (Timanian?) basement fabrics, and ( **B**) early Cenozoic formation of the Petuniabukta Syncline and Eurekan reactivation and/or overprint of (Timanian?) basement fabrics and Devonian to Carboniferous normal faults dominantly as top-west and top-SSW thrusts and deposition of lower Cenozoic sedimentary strata in the Central Tertiary Basin south of major NNE-dipping and west of major east-dipping basement-seated shear zones and inverted Devonian to Carboniferous normal faults. Left-lateral offset of the Balliolbreen Fault by NNE-dipping Devonian normal faults may have occurred in the Devonian to Mississippian due to normal-sinistral oblique-slip movements ( **A**) and/or in early Cenozoic times due to sinistral-reverse inversion of Devonian to Carboniferous normal faults ( **B**). Abbreviations: AA – Atomfjella Antiform; Af – Adolfbukta fault; BF – Balliolbreen Fault; CF – Cowantoppen Fault; EF – Ebbabreen Faults; GaF – Garmaksla fault; GF – Gipshuken Fault; HF – Hugindalen Fault; KF – Kampesteindalen Fault; OF – Odellfjellet Fault; ORS – Old Red Sandstone; OvF – Overgangshytta fault; PS – Petuniabukta Syncline; TGFZ – Triungen–Grønhorgdalen Fault Zone.

The overall N–S-trending ridge in southern Billefjorden ( Figure 3D) was interpreted as the uplifted (i.e., inverted) hanging wall of NNE–SSW-striking fault segments of the Billefjorden Fault Zone (most likely the Balliolbreen Fault) and/or as east-dipping carbonate beds of the Wordiekammen Formation. The ridge is offset left-laterally and drag-folded by WNW–ESE-trending fault-related escarpments on bathymetric data that correlate with early Cenozoic Eurekan thrusts overprinting and/or that formed parallel to NNE-dipping, inverted, oblique-slip sinistral-normal Devonian to Carboniferous faults ( Figure 4A–B). This suggests that the Billefjorden Fault Zone and the Billefjorden Trough are segmented by sub-orthogonal oblique-slip faults and shear zones, most likely since the Early Devonian ( Figure 5A), which is supported by a 410 Ma U–Th–Pb age for sinistral strike-slip movements along NW–SE-striking mylonitic shear zones in Proterozoic basement rocks in Oscar II Land ( Ziemniak *et al*., 2020), and possibly earlier as suggested by the basement-seated character of the NNE-dipping mylonitic shear zone ( Figure 4A–B). Segmentation is also supported by numerous WNW–ESE-trending fault-related escarpments on satellite images and in the field within Lower Devonian rocks of the Wood Bay Formation in Brimerpynten and Narveneset (electronic supplement 10 and 11).

Segmentation by WNW–ESE-striking faults would explain the gently SW- to south-dipping attitudes of bedding surfaces in Lower Devonian strata of the Wood Bay Formation in Triungen ( Dallmann *et al*., 2004b and electronic supplement 14) through syn-sedimentary Devonian down-faulting to the north along a NNE-dipping fault analogous to those observed on seismic data in Billefjorden ( Figure 4A–B). The presence of inverted Devonian NNE-dipping faults in Billefjorden further accounts for anomalous subvertical, E–W-trending bedding surfaces in the Wood Bay Formation in southern Mimerdalen on the northern slope of Yggdsrasilkampen (i.e., suborthogonal to elsewhere in Mimerdalen; Dallmann & Piepjohn, 2020) through a combination of Early Devonian, down-NNE, syn-sedimentary normal faulting and southwards tilting, and subsequent top-SSW Eurekan folding and inversion and/or overprinting along (a) NNE-dipping fault(s) like those observed on seismic data in Billefjorden ( Figure 4A–B) and onshore northwestern Spitsbergen ( Friend *et al*., 1997, figure 12B–C; McCann, 2000). This interpretation is supported by occurrences of subvertical, E–W- to WNW–ESE-trending bedding surfaces within Lower Devonian sedimentary rocks of the Wood Bay Formation in the fjord ( Figure 4A–B and dotted white lines in Figure 4G).

Segmentation of the N–S-trending Billefjorden Trough may explain the significant thickness variations of coal-rich sedimentary deposits of the Billefjorden Group in central Spitsbergen. This stratigraphic unit, which comprises thickened coal seams in Pyramiden ( Livshits, 1966) and Brucebyen ( Aakvik, 1981; Cutbill *et al*., 1976), is very thin (<50 m) to absent in Yggdrasilkampen in the south ( Dallmann *et al*., 2004a), and thinner northwards in Elsabreen and De Geerfjellet ( Aakvik, 1981, figure 8.1.3; Cutbill *et al*., 1976; Gjelberg, 1984). Notably, in Yggdrasilkampen, very thin portions of the Billefjorden Group succession are unconformably overlain by Upper Pennsylvanian to lowermost Permian sedimentary strata of the Wordiekammen Formation ( Dallmann *et al*., 2004a; Koehl *et al*., 2022; Manby *et al*., 1994). Whether uppermost Devonian to Mississippian sedimentary strata of the Billefjorden Group were deposited and subsequently eroded prior to the latest Mississippian or never or partly deposited because this area was exposed to continental erosion, a potential explanation for such thinning of the succession in Yggdrasilkampen and for the limited extent of thick coal-rich deposits in Pyramiden might be normal movements along (a) NNE-dipping brittle fault(s) downthrowing the Pyramiden block to the north during the latest Devonian to Middle Pennsylvanian, i.e., prior to the deposition of the Wordiekammen Formation, which is of comparable thickness in Pyramiden and Yggdrasilkampen ( Dallmann *et al*., 2004a). This interpretation is supported by the presence of several NNE-dipping faults in Billefjorden such as the Garmaksla fault, which bounds a NNE-thinning, wedge- to fan-shaped unit of uppermost Mississippian to Lower Pennsylvanian sedimentary rocks of the Hultberget and Ebbadalen formations in northern Billefjorden and is therefore believed to have accommodated normal movement at that time ( Figure 3D and Figure 4A–B).

Segmentation of the Billefjorden Trough by WNW–ESE-striking fautls is further supported by the calculated surface slope angle (in a critical wedge taper; e.g., Dahlen, 1990) between outcrops of the base Wordiekammen Formation in Pyramiden and Yggdrasilkampen (c. 6.50°), which is 3.5 times higher than between Yggdrasilkampen and Asvindalen farther south (c. 1.99°; electronic supplement 13) where the contact crops out at sea level ( Dallmann *et al*., 2004a). The calculated angle suggests that the uplift of the base Wordiekammen Formation in Pyramiden (located at an altitude of c. 850 m, i.e., higher than the c. 350 to 400 m altitude in Yggdrasilkampen) may be related to top-SSW early Cenozoic movements (folding; Figure 4A–B). Of the c. 475 m high southward down-drop in altitude of the base of the Wordiekammen Formation, about 145 m are related to the local 1.99° dip angle. This indicates that c. 330 m of the southward down-drop are imputable to top-SSW Eurekan folding in Mimerdalen (electronic supplement 13).

Thickening of uppermost Devonian to Mississippian sedimentary strata of the Billefjorden Group in Birger Johnsonfjellet, Petuniabukta, and Triungen, and thinning farther north in Faraofjellet and Citadellet ( Aakvik, 1981; Cutbill *et al*., 1976, figures 8.1.1–8.1.3; Verba, 2013, exploration well 116) may indicate further segmentation of the Billefjorden Trough and an architecture consisting of at least two WNW–ESE-trending, latest Devonian to Mississippian (to Early to Middle Pennsylvanian?), minor horsts and grabens interfering with the main N–S-trending half graben. The southern graben may extend from Brucebyen to Pyramiden and is bounded to the north by the SSW-dipping Adolfbukta fault ( Figure 3B and Koehl *et al*., 2023b, figures 4a and d–e, and 5b) and to the south by the NNE-dipping Garmaksla fault ( Figure 4A–B). The northern graben may extend from Petuniabukta and Birger Johnsonfjellet to Triungen and is bounded to the south by the NNE-dipping Hugindalen Fault and, to the north, either by the SW-dipping Ebbabreen faults ( McCann & Dallmann, 1996; Piepjohn *et al*., 1997; Piepjohn, 2000) or by the SSW-dipping McCabefjellet fault zone ( Koehl *et al*., 2023b).

Segmentation of the Billefjorden Trough is also suggested by the presence of Proterozoic basement rock crosscut by abundant WNW–ESE-striking fault-related escarpments west of the speculated trace of the Balliolbreen Fault in Ferdinandbreen ( Koehl *et al*., 2023b, figure 5C). This outcrop of Proterozoic basement rocks is located within the possible horst structure bounded by the WNW–ESE- to E–W-striking (normal) Hugindalen and Adolfbukta faults ( Figure 1B; see also Piepjohn, 2000, figure 3), and it is therefore possible that the Balliolbreen Fault is laterally and/or vertically offset by both horst-bounding faults.

### Implications for post-Caledonian extension in central and northwestern Spitsbergen

Interpretation of seismic data and depth conversion in Billefjorden, and earlier field studies in Billefjorden and seismic interpretation in Sassenfjorden and Tempelfjorden support kilometer-scale (up to 4.35 km) down-NNE normal displacement along NNE-dipping faults and shear zones. Normal movement was coeval with the deposition of Lower Devonian to Early (Middle?) Pennsylvanian strata of the Siktefjellet, Red Bay, and Andrée Land groups ( Figure 4A–B), Billefjorden Group ( Koehl, 2021, e.g., figures 4A–B), and (lower) Gipsdalen Group (at least Hultberget Formation and lower part of the Ebbadalen Formation; Figure 4A–B). This is shown by the presence of large, fault-bounded, wedge- to fan-shaped seismic units thinning towards the north ( Figure 4A–B; Koehl, 2021, e.g., figures 4A–B). Previous field studies in Lower Devonian sedimentary rocks onshore northernwestern Spitsbergen already suggested that sediments from the Siktefjellet and Red Bay groups and of the Wood Bay Formation were sourced from the south ( Dallmann & Piepjohn, 2020; Friend *et al*., 1966; Friend & Moody-Stuart, 1972; Murascov & Mokin, 1979) and deposited along NNE-dipping normal faults through kilometer-scale normal movements ( McCann, 2000), thus supporting our datasets ( Figure 5A).

Recently, Braathen *et al*. (2018) argued for significant top-north (> 50 km?) movements along the Keisarhjelmen Detachment in northwestern Spitsbergen based on the consistent SSW-tilting of strata of the Siktefjellet and Red Bay groups and on kilometer-scale normal offsets along NNE-dipping faults (some of which are thought to root into the Keisarhjelmen Detachment) showing slickensides indicating top-NNE normal movements ( Friend *et al*., 1997; McCann, 2000). Such interpretation is strongly disputed by Dallmann & Piepjohn (2018), mostly because some (most?) E–W- to WNW–ESE-striking faults seem to crosscut and left-laterally offset Proterozoic basement rocks of the Bockfjorden Anticline (e.g., Gee, 1972) instead of rooting into the Keisarhjelmen Detachment. Analogously to Braathen *et al*. (2018), McCann (2000) suggested that NNE-dipping normal faults in Haakon VII Land accommodated c. 30 km of north–south extension based on up to 45° SSW-tilting of Devonian rotated fault-blocks. However, inversion of analogous Devonian normal faults and the presence of numerous early Cenozoic top-SSW thrusts in equivalent Lower Devonian strata in Billefjorden ( Figure 4A–H) suggest that NNE-dipping Devonian normal faults in Haakon VII Land may have been inverted and that Lower Devonian deposits were (at least partly) reworked (i.e., further tilted) by early Cenozoic top-SSW thrusting. This is also supported by the proximity of NNE-dipping faults in Haakon VII Land to the West Spitsbergen Fold-and-Thrust Belt, i.e., much closer to the collision zone between Greenland and Svalbard than their equivalents in Billefjorden, the latter of which were extrensively reworked by Eurekan deformation ( Figure 4A–H). Thus, it is conceivable that the steep SSW-tilt of Lower Devonian strata in Haakon VII Land is actually the product of Devonian normal block-faulting and superimposed early Cenozoic thrusting ( Figure 7 and Friend *et al*., 1997, figure 12B–C), i.e., that the amount of north–south extension suggested by McCann (2000) and Braathen *et al*. (2018) was overestimated.

### Implications for Svalbardian and Eurekan deformation events

Seismic and bathymetric data show the occurrence of major vertical and lateral offsets of stratigraphic units and possibly of the Balliolbreen Fault (if present at all; see also Koehl, 2021 and Koehl *et al*., 2022 for discussion of the onshore trace of the Balliolbreen Fault) across major NNE-dipping inverted Devonian normal faults and related top-SSW décollements and thrusts with ramp anticline and contractional duplexes in southern Billefjorden ( Figure 3D & Figure 4A–H). Since many of these thrusts and duplexes crosscut Permian strata of the Wordiekammen and Gipshuken formations, they most likely formed during Eurekan contraction in the early Cenozoic. It is conceivable that some of the observed contractional structures in Lower Devonian strata represent Svalbardian structures, but it is not possible to distinguish these from Eurekan structures. However, based on the geometrical similarities and on comparable amounts of top-SSW offset along contractional structures in both Lower Devonian and lower Permian strata in Billefjorden, it is more probable that all these structures formed together in the early Cenozoic. A synchronous formation of all contractional structures is also supported by the possible (hard to soft) linkage of deep low-angle thrusts in Lower Devonian rocks with analogous shallow thrusts crosscutting strata of the Wordiekammen and Gipshuken formations by aggregates of top-SSW contractional duplexes ( Figure 4A–B). This is further supported by a revision of the ages of multiple stratigraphic units and a reinterpretation of the significance of Late Devonian–Mississippian geochronological ages suggesting that the Svalbardian Orogeny did not occur in Svalbard (see discussion and references in Koehl *et al*., 2022b).

The dominant WNW–ESE strike and top-SSW (and subsidiary top-NNE) transport direction of Eurekan décollements, and contractional duplexes and folds within Devonian to Permian sedimentary strata in southern Billefjorden ( Figure 4A–B and electronic supplements 3 and 4) diverge from the dominant N–S-striking Eurekan gra in Spitsbergen (e.g., Dallmann *et al*., 1993; Haremo & Andresen, 1992; Haremo *et al*., 1990; Harland *et al*., 1974). Nonetheless, WNW–ESE-trending Eurekan structures exist both in central (e.g., top-SW inversion of the Cowantoppen Fault; Harland *et al*., 1974; Figure 1B) and western Spitsbergen (e.g., in Brøggerhalvøya; Figure 1A). Notably, in Brøggerhalvøya, Eurekan contraction resulted in the formation of low-angle top-NNE thrusts with imbricate-fan geometries bounding hundreds of meter-thick thrust sheets, and forming contractional duplexes and antiformal stacks with bedding-parallel décollements and detachments localized along rheological boundaries and within weak sedimentary beds, e.g., carbonate and evaporitic succession of the Gipshuken Formation ( Bergh *et al*., 2000; Piepjohn *et al*., 2001; Saalmann & Brommer, 1997; Saalmann *et al*., 1997; Saalmann & Thiedig, 2000; Saalmann & Thiedig, 2001). In addition, top-SSW imbricate thrusts in Blomstrandhalvøya showing comparable geometries and sizes to Eurekan thrusts in southern Billefjorden ( Figure 4A–B & D–H) and initially ascribed to latest Devonian Svalbardian contraction ( Buggisch *et al*., 1994; Kempe *et al*., 1997; Thiedig & Manby, 1992) likely formed in the early Cenozoic ( Koehl, 2020b; Koehl *et al*., 2022b). Another similar structure is the NNE-dipping Overgangshytta fault in Odellfjellet (central–northern Spitsbergen), which formed or was reactivated as a top-SSW Eurekan thrust ( Koehl & Muñoz-Barrera, 2018).

Lower Devonian sedimentary rocks of the Siktefjellet, Red Bay and Andrée Land groups in southern Billefjorden are considerably more deformed than strata of the Hultberget, Ebbadalen and Minkinfjellet formations and experienced a more intense early Cenozoic reworking than adjacent and underlying Proterozoic basement rocks as shown by the significantly higher number of Eurekan thrust and duplex structures within Lower Devonian strata ( Figure 4A–H). This suggests that the thick (maximum c. 3.85 to 4.06 km) Lower Devonian sedimentary rocks may have acted as a buffer and localized the formation of most contractional structures during Eurekan tectonism. Hence Lower Devonian rocks (partially) decoupled basement rocks, which are crosscut by gently dipping basement-seated mylonitic shear zones, from overlying sedimentary rocks, which are truncated by low-angle brittle thrusts. Eurekan deformation was therefore partitioned between the intensely deformed belt of Lower Devonian rocks in the south and poorly deformed Pennsylvanian to Permian sedimentary units in the north ( Figure 4A–H and electronic supplement 7). If strain decoupling and partitioning of Eurekan deformation occurred within Lower Devonian sedimentary successions in the Billefjorden area, this process is also very likely to have occurred elsewhere in Spitsbergen and the Barents Sea where Devonian to Mississippian successions are up to 8.6 to 9.675 km thick and most likely of similar composition ( Friend *et al*., 1997; Murascov & Mokin, 1979). Strain partitioning of Eurekan deformation in Devonian to Mississippian sedimentary rocks notably occurred in Pyramiden, Sassenfjorden–Tempelfjorden ( Koehl, 2021), and Garmdalen in central Spitsbergen (e.g., top-west thrusts localized within weak coals of the Billefjorden Group, Balliolbreen Fault flattening into a bedding-parallel décollement, and brecciated unconformities between the Wood Bay Formation, the Billefjorden Group and the Wordiekammen Formation; Manby *et al*., 1994, their Figure 12; Koehl *et al*., 2022, their Figure 2), and in Adriabukta in southern Spitsbergen ( Koehl, 2020b; Koehl *et al*., 2022b). This strongly suggests that the Svalbardian Orogeny is not required to explain the strong deformation differences between intensely deformed Lower Devonian rocks and relatively undeformed Carboniferous to Permian strata in central Spitsbergen.

More specifically, contractional deformation intensity within thick Lower Devonian sedimentary rocks of the Siktefjellet and/or Red Bay and Andrée Land groups varies greatly. On the one hand, these units are crosscut by abundant thrusts and contractional duplexes and tightly folded in the lower part, which consists of highly heterogeneous (interbedded conglomerate, sandstone, siltstone, and mudstone; Friend *et al*., 1997; Gee & Moody-Stuart, 1966; McCann, 2000; Murascov & Mokin, 1979) deposits of the Siktefjellet and/or Red Bay groups. On the other hand, relatively homogeneous upper stratigraphic intervals like the Wood Bay Formation ( Dallmann & Piepjohn, 2020; Friend *et al*., 1966; Friend & Moody-Stuart, 1972; Murascov & Mokin, 1979) are only mildly deformed into open upright folds, e.g., wedge- to fan-shaped alluvial fan deposits ( Figure 4A–B). The numerous pronounced lithological heterogeneities in the Siktefjellet and Red Bay groups likely facilitated the localization of duplex structures bounded by roof- and floor-thrusts acting as décollements within weak shaly units ( Figure 4G) and, thus, accommodated extensive amounts of deformation in the early Cenozoic compared to overlying more homogeneous deposits (i.e., strain partitioning and decoupling).

Early Cenozoic strain decoupling in Billefjorden is also particularly well illustrated by low-angle Eurekan thrusts merging downwards with a local décollement with detached ramp anticline along the stratigraphic boundary between the Wood Bay and Wordiekammen formations, which decoupled the Upper Pennsylvanian to lower Permian overburden from the Lower Devonian sedimentary successions during Eurekan deformation ( Figure 4A–B & F), i.e., further suggesting that Svalbardian deformation is not needed to explain (inter-stratigraphic) variations in deformation patterns and intensity in central Spitsbergen. Early Cenozoic décollements at the base of the Wordiekammen Formation explain the presence of top-west folds and thrusts in deposits of the Billefjorden Group, Hultberget and Ebbadalen formations ( Koehl *et al*., 2022), and of the Minkinfjellet Formation ( Senger *et al*., 2018), whereas unconformably overlying strata of the Wordiekammen Formation in Garmdalen and Lykteneset are apparently undeformed (flat-lying; see Figure 1B for location).

In addition, recent analysis of seismic data in Sassenfjorden and Tempelfjorden showed the presence of similar WNW–ESE- and N–S-striking, top-SSW to top-NNE and top-west early Cenozoic Eurekan thrusts in sedimentary strata of the Billefjorden and Gipsdalen groups ( Koehl, 2021, figure 4A–F). Top-SSW thrusts are restricted to the Hultberget, Ebbadalen, Minkinfjellet and Wordiekammen formations, whereas top-NNE and top-west thrusts occur dominantly within lower Permian strata of Gipshuken Formation. The former flatten downwards and sole into thin uppermost Devonian to Mississippian coal-rich strata of the Billefjorden Group known for their weak behavior and propensity to localize deformation ( Koehl, 2021, figure 3B & 4B), and the latter into the stratigraphic boundary between the Wordiekammen and Gipshuken formations ( Koehl, 2021, figure 4A–C). These observations therefore suggest the presence of bedding-parallel décollements in at least two stratigraphic levels in this area and, thus, a strong influence of strain decoupling in central Spitsbergen during Eurekan deformation. Such a strong effect of strain partitioning was predicted earlier (but not documented) by critical wedge taper models by Braathen *et al*. (1999).

Seismic and bathymetric data in Billefjorden show that numerous Eurekan décollements, thrusts, contractional duplexes, and folds strike WNW–ESE and accommodated dominant sinistral-reverse, top-SSW (and subsidiary top-NNE) movements ( Figure 4A–H, and electronic supplements 3 and 4). Some of these thrusts and inverted Devonian to Carboniferous normal faults (and possibly related mylonitic shear zone at depth) accommodated hundreds of meter- to kilometer-scale (950 to 2350 m cumulated) top-SSW reverse offset of the Top Basement reflection as shown by the abrupt southwards deepening of this reflection across multiple faults from c. 2540 to 2850 m depth (i.e., ca. 1.1 s TWT) in the hanging of the northermost NNE-dipping fault to a depth of c. 4780 to 4920 m (i.e., ca. 1.4 s TWT) below the thickest portion of Lower Devonian sedimentary deposits in Billefjorden ( Figure 4A–B and electronic supplement 6). Such large offsets along WNW–ESE-striking faults suggest that other N–S kilometer-scale variations in the depth of the top of the Proterozoic basement in Billefjorden may, as well, be related to top-SSW movements along yet-to-be-mapped, NNE-dipping (Eurekan thrust and/or inverted Devonian to Carboniferous normal) faults. Notably, the southward deepening of Proterozoic basement rocks from a 1290 m depth in Petuniabukta (well 116 of Trust Arktikugol; Senger *et al*., 2019; Verba, 2013) to a depth of c. 2540 to 2850 m near Pyramiden, where the seismic line shown in Figure 4A–B terminates, suggests as much as c. 1250 to 1550 m of combined top-SSW Eurekan reverse offset along NNE-dipping faults and/or down-SSW normal movements along potential Devonian to Carboniferous faults between these two areas, including at least 300 to 480 m down-SSW movements along SSW-dipping normal faults (e.g., Adolfbukta and Kampesteindalen faults). This is further supported by the occurrence of top-SSW Eurekan and/or NNE-dipping, inverted Devonian–Carboniferous faults in Odellfjellet (e.g., Overgangshytta fault; Koehl & Muñoz-Barrera, 2018).

Eurekan thrusts and décollements, and Devonian–Carboniferous faults (and related mylonitic shear zones?) inverted in the early Cenozoic typically show 500 to 2000 m wide sinistral displacement of upper Paleozoic stratigraphic units including the Wordiekammen Formation ( Figure 3D and Figure 4A–B and electronic supplement 9). Although recent geochronological studies in western Spitsbergen suggest that sinistral strike-slip movements along WNW–ESE-striking shear zones occurred in the Early Devonian ( Ziemniak *et al*., 2020; Figure 5A), the left-lateral offsets of east-dipping strata of the Wordiekammen Formation and their involvement into sinistral drag-folding indicate that sinistral movements also occurred along WNW–ESE-striking faults and shear zones in the early Cenozoic ( Figure 5A–B).

**Figure 6.**
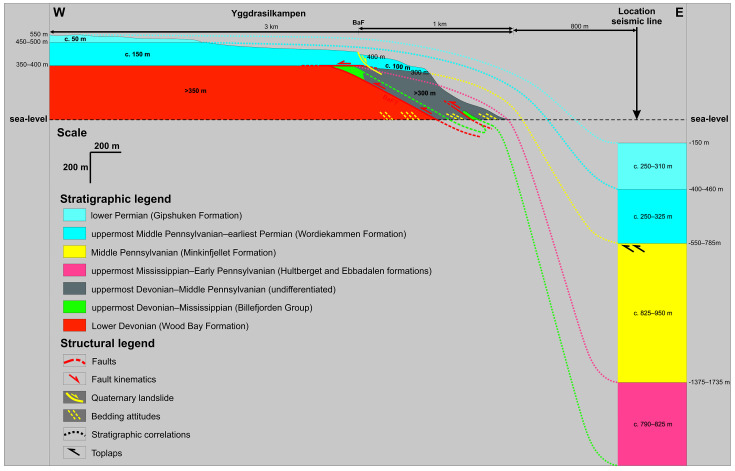
Onshore–offshore correlation between Garmdalen and Lykteneset and seismic interpretation in the hanging wall of the Garmaksla fault in nearshore fjord areas (see Figure 4A–B for seismic interpretation). The geology of onshore areas is based on Manby *et al.* (1994) and Koehl *et al.* (2022). Note that vertical and horizontal scales are the same.

The eastward dip of stratigraphic boundaries and bedding surfaces onshore western Billefjorden ( Dallmann *et al*., 2004a; Koehl *et al*., 2022) and in the fjord ( Figure 3D and electronic supplement 9) is most likely related to top-west early Cenozoic folding and thrusting along the Balliolbreen Fault and related faults ( Figure 6). Top-west Eurekan structures and east-dipping strata are known from onshore areas in Billefjorden ( Harland *et al*., 1988; Koehl, 2021; McCann & Dallmann, 1996; Ringset & Andresen, 1988) and nearshore areas in Sassenfjorden–Tempelfjorden ( Koehl, 2021).

**Figure 7.**
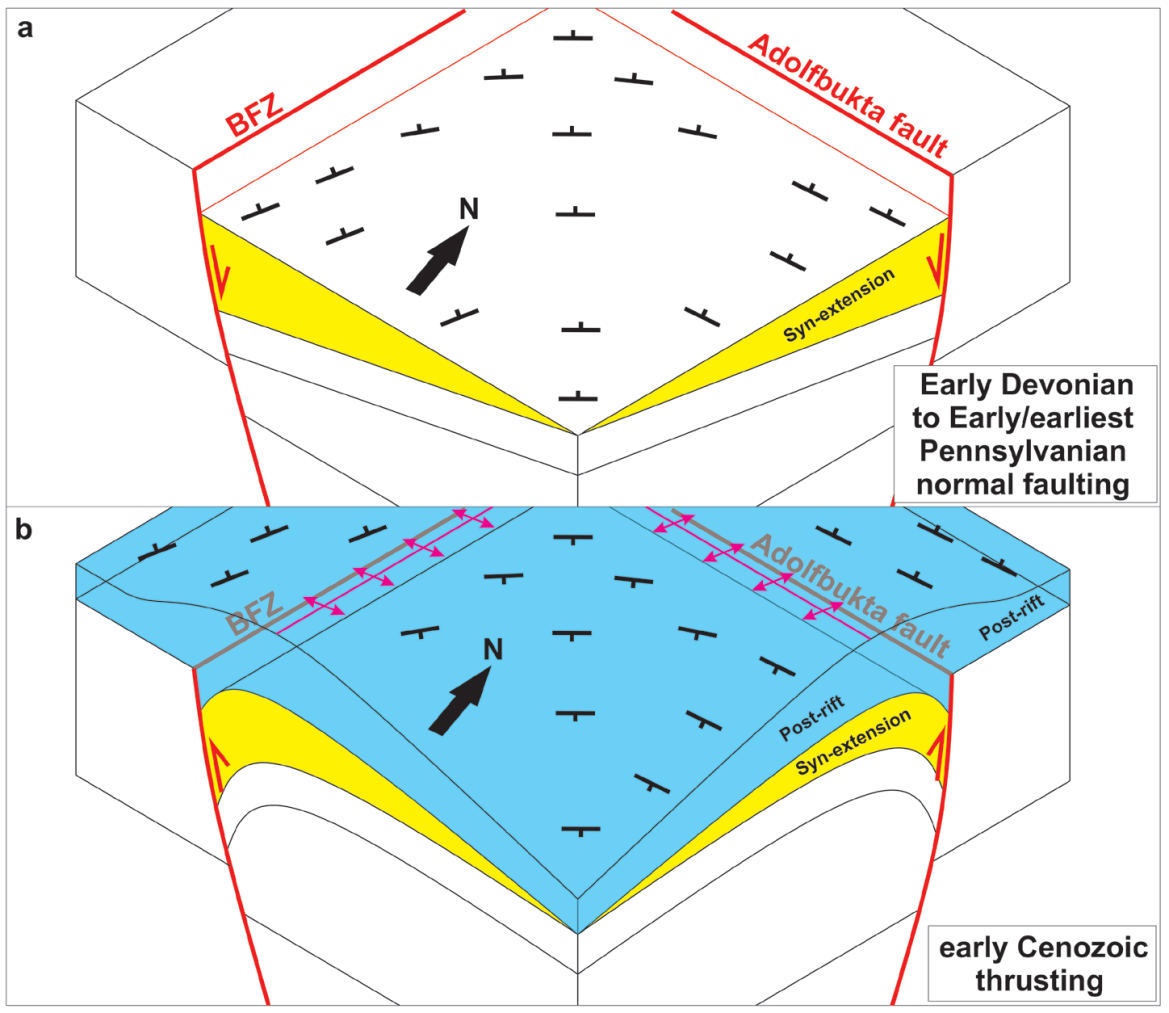
Schematic sketches showing ( **A**) tilting of syn-tectonic, Early Devonian to earliest/Early Pennsylvanian sedimentary strata along coeval ESE- and SSW-dipping normal faults (red lines), and ( **B**) back-tilting overall to the east-southeast to southeast due to inversion of post-Caledonian normal faults during early Cenozoic Eurekan deformation. Notice the formation of kilometer-wide, open folds above major inverted normal faults (fuchsia lines). Black symbols reflect bedding attitude. Abbreviations: BFZ – Billefjorden Fault Zone. Modified after Koehl *et al.,* 2023a (originally published under CC-BY 4).

### Implications for the Petuniabukta Syncline

In the area of Pyramiden, Elsabreen and Svenbreenhøgda, the top-basement unconformity and sedimentary strata of the Ebbadalen Formation ( Braathen *et al*., 2011) and of the Minkinfjellet Formation dip gently to the east-southeast to southeast, and graben structures within the Minkinfjellet Formation just east of Pyramiden are tilted east-southeastwards to southeastwards ( Koehl *et al*., 2016; Koehl *et al*., 2023a). Braathen *et al*. (2011) ascribed these southeastward to east-southeastward dips and tilts to the presence of a SE- to SSE-dipping relay zone between the Balliolbreen and Odellfjellet faults in Pyramiden and Elsabreen, possibly including the Pyramiden Fault ( Smyrak-Sikora *et al*., 2018). However, the dip of strata of the Ebbadalen Formation does not appear to change across (i.e., because of) this fault ( Smyrak-Sikora *et al*., 2018, figure 7). Another possibility is that the observed dip and tilts are related to early Cenozoic inversion of both N–S- and WNW–ESE-striking faults such as the east-dipping Balliolbreen and Odellfjellet faults and the SSW-dipping Adolfbukta fault. For example, early Cenozoic reverse reactivation of the east-dipping segments of the Billefjorden Fault Zone may have tilted Pennsylvanian sedimentary strata of the Ebbadalen and Minkinfjellet formations (dominantly) to the east and superimposed (preceding, simultaneous or subsequent) reverse movement along the SSW-dipping Adolfbukta fault at depth would have resulted in an overall east-southeastward to southeastward tilt of the strata and graben structures ( Figure 7). Alternatively, the ESE-dip of Pennsylvanian sedimentary strata in Pyramiden, Elsabreen and Svenbreenhøgda might be related to the actual strike of fault segments of the Billefjorden Fault Zone being NNE–SSW instead of N–S to NNW–SSE ( Figure 3D; Koehl *et al*., 2023a; Koehl *et al*., 2023b), and to tilting of the strata during Eurekan inversion of NNE–SSW-striking faults segments of the Billefjorden Fault Zone (and minor folding). This is supported by the similar dip of uppermost Devonian to Mississippian sedimentary strata of the Billefjorden Group and by the dominant top-WNW sense of shear of early Cenozoic Eurekan contractional duplexes and thrust faults (which are possibly part of the Balliolbreen Fault) within this stratigraphic unit in Pyramiden ( Koehl, 2021).

In southern Billefjorden, the Gipshuken Formation crops out at sea level onshore Kapp Fleur de Lys and Anservika ( Dallmann *et al*., 2004a; Dallmann, 2015; Harland *et al*., 1974), and close to the surface in the fjord between Kapp Fleur de Lys and Anservika ( Figure 4A–B). However, farther north, this stratigraphic unit crops out at up to 450 to 500 m altitude in Yggdrasilkampen (west of the Billefjorden Fault Zone), at an altitude of 600 m east of the Billefjorden Fault Zone in Campbellryggen ( Figure 1B; Dallmann *et al*., 2004a), and at a depth of c. 150 to 310 m in the fjord ( Figure 4A–B). This configuration reflects an overall south to southwestwards dip of these sedimentary strata onshore on both sides of the fjord, as well as a 600 to 900 m negative topographic relief at Gipshuken Formation level in the fjord between Yggdrasilkampen and Campbellryggen. This suggests that, in addition to being offset by (up to 200 m of) top-west reverse movement along the Gipshuken segment of the Billefjorden Fault Zone in Cowanodden and Gipsvika ( Dallmann *et al*., 2004a; Dallmann, 2015; Harland *et al*., 1974; see locations in Figure 1B), the Gipshuken Formation is either (1) offset with a top-west reverse sense of shear by (a) N–S- to NNE–SSW-striking fault(s) between the seismic section shown in Figure 4A–B and the eastern coastline of Billefjorden (e.g., the Odellfjellet Fault and/or Gipshuken Fault) or (2) by normal faults forming a (rectangular) mini-basin within the fjord, and/or (3) involved into a kilometer-wide, N–S- to NNE–SSW-trending, gently north- to northeastwards-plunging open syncline. The present analysis of bathymetric and seismic data in Billefjorden does not support the presence of any major N–S-striking fault in the fjord other than the Balliolbreen Fault ( Figure 3D and Figure 4A–B and electronic supplements 3 and 4). However, previous study of the kilometer-wide Petuniabukta Syncline indicates the occurrence of Eurekan contractional folding in northern Billefjorden ( Figure 5B; McCann & Dallmann, 1996). It is therefore most probable that the observed variations result from early Cenozoic folding related to the Petuniabukta Syncline ( Figure 6). This is further supported by the 600 to 900 m topographic relief at Gipshuken Formation level between Yggdrasilkampen and Campbellryggen, which is comparable to the >700 m relief defined by the Wordiekammen Formation in the Petuniabukta Syncline in Petuniabukta ( Maher & Braathen, 2011).

Similar topographic reliefs are recorded for the Top Wood Bay Formation and Top Wordiekammen Formation in the footwall of the Balliolbreen Fault in the south. There, the top of these formations crop out respectively at altitudes of c. 150 to 250 and c. 350 m onshore (in Nidedalen and Narveneset; see location in Figure 1B; Dallmann *et al*., 2004a; Dallmann & Piepjohn, 2020) and are located at respective depths of c. 720 to 860 and c. 470 to 530 m in the fjord (i.e., topographic reliefs of 870 to 1110 and 820 to 880 m; Figure 4A–B and electronic supplement 6). This suggests that synclinal folding continues in the footwall of the (offset) southern portion of the Balliolbreen Fault (i.e., south of the Garmaksla fault in Figure 4A–B) and, therefore, precludes that folding in this area was influenced by Carboniferous to Permian, normal-fault propagation folding ( Figure 5B). Since the Gipshuken Formation crops out at sea-level onshore Kapp Fleur de Lys and Anservika and reaches near-surface level within the fjord near these areas ( Figure 4A–B), the Petuniabukta Syncline most likely dies out southwards ( Figure 5B) or may, like the Billefjorden Fault Zone, be offset laterally by WNW–ESE-striking faults ( Figure 3D).

### Possible origin for WNW–ESE-striking faults and shear zones in Billefjorden

Seismic data in Billefjorden show a dominance of WNW–ESE-striking early Cenozoic Eurekan thrusts and (inverted) Devonian to Carboniferous normal faults. At depth, these faults merge and root into thick packages of sub-parallel moderate-amplitude reflections interpreted as mylonitic ductile shear zones and associated fabrics in adjacent and underlying Proterozoic basement rocks ( Figure 4A–B). These observations suggest strong control of preexisting WNW–ESE-trending basement grain on post-Caledonian Devonian to Carboniferous extensional faulting and their early Cenozoic inversion and overprinting.

The NNE-dipping basement-seated shear zones are highly oblique to sub-orthogonal to N–S-trending Caledonian fabrics. Considering evidence supporting the presence of WNW–ESE-striking Timanian ductile structures and fabrics in southwestern ( Gayer *et al*., 1966, their samples 49 and 50, and their hypotheses 1 and 2 also discussed in Harland *et al*., 1966; Majka *et al*., 2008; Manecki *et al*., 1998; Majka *et al*., 2012; Mazur *et al*., 2009), western ( Horsfield, 1972), northwestern ( Gayer *et al*., 1966, their samples 53 and 60; Gromet & Gee, 1998; Koglin *et al*., 2022; Ohta *et al*., 2003; Peucat *et al*., 1989), and northeastern Spitsbergen ( Gayer *et al*., 1966; Hamilton & Sandford, 1964, their samples 19–22; Johansson *et al*., 2004; Johansson *et al*., 2005), such as the Vimsodden–Kosibapasset Shear Zone ( Faehnrich *et al*., 2020; Mazur *et al*., 2009, their sample 16-73A), we propose that WNW–ESE-striking basement-seated mylonitic shear zones and fabrics in southern Billefjorden formed during the Timanian Orogeny ( Figure 4A–B). Recent studies reveal the presence of deep, crustal-scale, WNW–ESE- to NW–SE-striking shear zones and thrust systems in the northern Barents Sea, Svalbard and Storfjorden that merge with Timanian faults in northwestern Russia ( Klitzke *et al*., 2019; Koehl, 2019; Koehl, 2020a; Koehl *et al*., 2022a). Since WNW–ESE-striking faults and fabrics in basement rocks in Billefjorden align with and strike parallel to some of the main Timanian thrusts and shear zones mapped in northern Storfjorden and Sassenfjorden ( Koehl *et al*., 2022a, e.g., their Kongsfjorden–Cowanodden fault zone), it is probable that they are part of the same fault system. A Timanian origin was also proposed for WNW–ESE-striking faults in Proterozoic basement rocks in Mittag-Lefflerbreen and potential late Paleozoic to early Cenozoic overprints in Odellfjellet (e.g., Overgangshytta fault; Koehl & Muñoz-Barrera, 2018).

Furthermore, it is probable that a major, crustal-scale, WNW–ESE-trending, basement-seated zone of weakness exists at depth, and that this zone separates northern from southern Spitsbergen and is responsible for the local dominance of WNW–ESE-trending structures. This is based on the presence of numerous and dominant WNW–ESE-striking brittle faults and shear zones within Proterozoic basement rocks and Lower Devonian to lower Cenozoic sedimentary strata in central ( Figure 3A–D & Figure 4A–B and electronic supplements 3 and 4; Koehl *et al*., 2023a, figure 3a–b; Koehl *et al*., 2023b, figure 2, 3, 4a and d–e, and 5) and western Spitsbergen ( Bergh *et al*., 2000; Piepjohn *et al*., 2001; Saalmann & Brommer, 1997; Saalmann *et al*. 1997; Saalmann & Thiedig, 2000; Saalmann & Thiedig, 2001). It is also supported by the WNW–ESE-trending alignment of outcrops of uppermost Devonian to Permian sedimentary rocks of the Billefjorden and Gipsdalen groups between Dickson Land and Brøggerhalvøya (including in James I Land), and by the alignment of major WNW–ESE-striking faults in Billefjorden and Sassenfjorden (present contribution and Koehl, 2021) with major WNW–ESE-striking faults in Brøggerhalvøya. Such a deep weakness zone was previously suggested in western Spitsbergen by Harland & Horsfield ( 1974; e.g., Kongsvegen Fault and Lappsdalen Thrust), Harland & Wright ( 1979; e.g., Kongsvegen Fault Zone and Central-West Fault Zone) and Harland *et al*. ( 1993; e.g., Kongsfjorden–Hansbreen Fault Zone), though with various extents, trends, and geometries. This major zone of weakness is referred to as the NNE-dipping Kongsfjorden–Cowanodden fault in ongoing works ( Koehl, 2019; Koehl, 2020a; Koehl *et al*., 2022a).

## Conclusions

1) The several kilometer-thick successions of Lower Devonian sedimentary strata in Billefjorden and southeastern Dickson Land were deposited along syn-sedimentary WNW–ESE-striking faults comparable to faults in northwestern Spitsbergen.
2) The Billefjorden Trough and associated major N–S- to NNE–SSW-striking faults, like the Billefjorden Fault Zone, are segmented and offset by major WNW–ESE-striking faults, like the Adolfbukta fault, forming trough-oblique systems of grabens and horsts that localized the deposition of thickened coal-rich deposits of the Billefjorden Group during synchronous evolution of N–S- to NNE–SSW- and WNW–ESE-striking normal faults.
3) Eurekan strain partitioning and decoupling by thick Lower Devonian sedimentary successions acting as a weak buffer, the involvement of both Devonian and post-Devonian rocks in contractional deformation, and bedding-parallel décollements show that Late Devonian Svalbardian deformation is not required to explain differential deformation between folded Devonian and relatively undeformed Carboniferous to Permian rocks in Billefjorden.
4) The N–S- to NNE–SSW-trending Petuniabukta Syncline formed during early Cenozoic Eurekan deformation.
5) WNW–ESE-striking faults in Proterozoic basement and post-Caledonian sedimentary rocks in Billefjorden are following and, in places, merge with preexisting Timanian shear zones.
6) The dominance of WNW–ESE-striking faults and fabrics in Proterozoic basement rocks and Lower Devonian to lower Cenozoic sedimentary rocks both in central and western Spitsbergen suggests the presence of a major WNW–ESE-trending zone of weakness extending from Billefjorden–Sassenfjorden to Kongsfjorden and potentially merging with Timanian thrust systems in Storfjorden and the northern Barents Sea.

## Data Availability

The source data used in this study is not available publicly as it is under license by third parties. Please see below descriptions of the data sources and the information required to request access to the data directly from the third parties. The bathymetric data analysed in study ( Figure 3A–D and electronic supplement 2) was sourced from: The Norwegian Mapping Authority: Access to the data for research purposes can be requested by contacting the Norwegian Mapping Authority at https://www.kartverket.no/en/about-kartverket/contact-us. The University Centre in Svalbard: Access to the data for research purposes can be requested by contacting the University Center in Svalbard at post@unis.no. The Two-Way Time (TWT) seismic data analysed in this study was sourced from the DISKOS (Norwegian National Data Repository for Petroleum Data) database in Billefjorden. Access to the data for research purposes can be requested by contacting the Norwegian Petroleum Directorate at https://www.npd.no/fakta/om-oss/kontakt-oss/. Velocity data were sourced from exploration well 7816/12-1 in Reindalspasset (discussed in Eide *et al*., 1991, and from Gernigon *et al*., 2018). Access to the data for research purposes can be requested by contacting the third party companies that own the data, namely: Equinor A.S.A. at https://www.equinor.com/about-us/contact-us and Store Norske Spitsbergen Kulkompani at https://www.snsk.no/kontakt/ansatte (in this case the authors contacted Malte Jochmann; malte.jochmann@snsk.no). DataverseNO: Replication Data for: Devonian–Carboniferous extension and Eurekan inversion along an inherited WNW–ESE-striking fault system in Billefjorden, Svalbard. https://doi.org/10.18710/UCRW4L. ( Koehl *et al*., 2023c). This project contains the following underlying data: 00_ReadMe.txt. Figure 1– Figure 7 (high resolution versions of the figures included in this manuscript, in jpg format. All copyright permissions granted). Supplement figures 2–4, 7–9, 10–14 (high-resolution versions of the supplementary figures included in the extended dataset, Koehl *et al*., 2023d, in jpg format. All copyright permissions granted). DataverseNO: Supplements for Devonian–Carboniferous extension and Eurekan inversion along an inherited WNW–ESE-striking fault system in Billefjorden, Svalbard. https://doi.org/10.18710/1WTNQB. ( Koehl *et al*., 2023d). This project contains the following extended data: 00_ReadMe.txt. Koehl_et_al._supplements.docx (supplementary information and data to the present contribution including an extended description of the late Paleozoic sedimentary successions in central Spitsbergen from the literature, uninterpreted versions of the figures, additional seismic data, and extended description and interpretation of the upper Paleozoic sedimentary successions on seismic data in Billefjorden, depth-converted seismic data and details about the depth-conversion process, additional bathymetric profiles in the fjord, satellite photographs, outcrop photographs, and an extended discussion of the implications of the present study for tectonic extension in the study area. All copyright permissions granted). Koehl_et_al._supplements.pdf (pdf version of the above-described document). Data are available under the terms of the Creative Commons Zero "No rights reserved" data waiver (CC0 1.0 Public domain dedication).
