# Supplementary material for: Devonian–Carboniferous extension and Eurekan inversion along an inherited WNW–ESE-striking fault system in Billefjorden, Svalbard
Source: Open Res Eur. 2023 Aug 4;3:124. [Version 1] doi: 10.12688/openreseurope.15936.1 (PMC10646348; doi:10.12688/openreseurope.15936.1)
Supplement: Peer review history

# Reviewer report: Reviewer response for version 1

**DOI:** 10.21956/openreseurope.17210.r34343

**Published:** 2023-10-12

**Authors:** Alexander Peace

**Affiliations:** 1 McMaster University, Hamilton, Ontario, Canada

This is a review of “Devonian–Carboniferous extension and Eurekan inversion along an inherited WNW–ESE-striking fault system in Billefjorden, Svalbard” by Jean-Baptiste P. Koehl *et al*. Overall, I found that the paper addresses an interesting topic but that it contains serious problems that in its current state make it unsuitable for indexing. Having said that I think that these issues can be fixed, and that the resultant manuscript will be a good contribution.

 One of my main issues with the manuscript is the description of the data and methods. I felt that this section was not adequate for readers to fully understand the interpretations and limitations of the work. First, the manuscript needs better figures showing the locations of the seismic data used. I think some of this information is on figure 3 but it isn’t clear. Also, the acquisition, processing and interpretation parameters are not described. For example, what approach was taken with interpretation? This prevents a reader from fully evaluating the resultant geological models presented. I don’t think just naming the software used is enough. Regarding the bathymetric data, what is meant by 'high resolution' as noted in the text?

 Also regarding methods, I feel that the results could have been bolstered by integration with the openly available gravity and magnetic data. This would allow the authors to place more constraints on the interpretations presented. Why didn’t the study utilise such potential field data? Has this work already been done?

 I found that generally the manuscript is longer than it needs to be. Some sections in particular are much too long and the main points are lost in the text. For example, the Geological Setting section gives an excellent overview of the area, but how necessary is all of this information for the study? On the other hand, the Methods section only contains two paragraphs and nowhere near enough detail is given (as described above). Also regarding these introductory sections, I felt that the scientific rationale behind the work wasn’t particularly clear, i.e. what was the purpose of the study? Similarly, the discussion and interpretation sections are also overly long in my opinion.

 Finally, I found that place names/locations are not well shown on figures for readers not familiar with the setting.

 Other points:

- Figure 1: Overall, this is a good and useful figure but is there a colour scale for part 1 of this figure?
- Figure 3: Why are different colour scales used for A and B but no colour scale is given for D and C? What I mean by this is that I am not sure which scale I should be reading for C and D. Also what is the highshade direction used?
- Figure 4: A better location map is needed for the seismic data, as noted above. It is unclear to me where this line is from. The text on legend is too small on this figure. The boxes on part a are very hard to see and read too against the seismic line. Also, the interpretation process leading to the yellow horizons shown needs describing further.
- Discussion, The Adolfbukta fault: I am not sure that papers from 1997 count as recent. Even the most 'recent' one cited (2011) is 12 years old! Despite being referred to as 'older' the personal communication is the most recent item here. 
- Figure 5 (and associated text): The figures that present the model are of good quality, but I don’t feel that the text explains the model sufficiently. It isn’t that the text isn’t long enough, more that the way it is explained is quite convoluted and overly long. I suggest refining the discussion so that it is more focused.

Is the study design appropriate and does the work have academic merit?

Partly

Is the work clearly and accurately presented and does it cite the current literature?

Partly

If applicable, is the statistical analysis and its interpretation appropriate?

Not applicable

Are all the source data underlying the results available to ensure full reproducibility?

Partly

Are the conclusions drawn adequately supported by the results?

Partly

Are sufficient details of methods and analysis provided to allow replication by others?

No

Reviewer Expertise:

Structural geology, tectonics, applied geophysics

I confirm that I have read this submission and believe that I have an appropriate level of expertise to state that I do not consider it to be of an acceptable scientific standard, for reasons outlined above.

# Response

**Published:** 2023-10-14

**Authors:** Jean-Baptiste Koehl

**Affiliations:** Geosciences, Universitetet i Oslo, Oslo, Oslo, Norway

**Reply to Alexander Peace**   Dear Sir, Madam, thank you very much for your input on the manuscript, it is highly appreciated. Here is our reply to your comments. We hope the changes we implemented improve the shortcomings of the manuscript highlighted by your comments and suggestions. Please do not hesitate to contact us shall this not be the case for some comments.  

**Comments by the reviewer**

Comment 1: One of my main issues with the manuscript is the description of the data and methods. I felt that this section was not adequate for readers to fully understand the interpretations and limitations of the work.

Response: Agreed. This is partly addressed by the sentences added to the methods chapter. The authors of the present manuscript stress the importance of the Extended data section (notably Supplement S5, which details the interpretation of the stratigraphy) in understanding the interpretation presented. In addition, a solution will be discussed with the journal editorial team to enlarge Figure 4a–b. The authors of the present manuscript are open to Splitting Figure 4 into 2 separate figures should it be required by the journal’s standards. This way, Figure 4a–b would be enlarged to an entire page and so would the new figure 5a–f (currently Figure 4c–h). Also see response to comment 2 for the addition of the entire database used in the interpretation.

Changes: Added “Supplementary data (e.g., calculations related to depth conversion and topographic variations, bathymetric profiles, and further field evidence and extended discussion) are available as Extended data.”. Awaiting decision by the editorial team to split Figure 4a–h into Figure 4a–b and Figure 5a–f or leave as is but anyway enlarge Figure 4a–b to a full page. Also added information on the bathymetric data to the method chapter: “(the resolution of the data is 10 m laterally and 5 m vertically)” and “resolution: 0.05 m in all directions;”.  

Comment 2: First, the manuscript needs better figures showing the locations of the seismic data used. I think some of this information is on figure 3 but it isn’t clear.

Response: Agreed.

Changes: Changed the names of the seismic lines in Figure 3 in the present manuscript into “Figure 4a–b” and “Supplement S3a & S4a” and “Supplements S3b & S4b”. In addition, the entire seismic database used was included in Figure 1a in the present manuscript and added “showing the database of seismic reflection data used for the present study. The map is” to the caption of Figure 1a.  

Comment 3: Also, the acquisition, processing and interpretation parameters are not described. For example, what approach was taken with interpretation? This prevents a reader from fully evaluating the resultant geological models presented. I don’t think just naming the software used is enough.

Response: Agreed. Many more details about the interpretation (notably of the stratigraphy) are included in Supplement S5 in the Extended data section. A direct tie was established with the nearby onshore geology, which is summarized extensively in Supplement S1. See also response to comment 1. Regarding the acquisition and processing of the seismic surveys used, these can be accessed from the DISKOS database of the Norwegian Petroleum Directorate, and are not the focus of the present manuscript.

Changes: See response to comment 1.  

Comment 4: Regarding the bathymetric data, what is meant by 'high resolution' as noted in the text?

Response: Agreed. High-resolution means 10 m by 10 m laterally and 5 m vertically for the bathymetric data of the Norwegian Polar Institute and 0.05 m in all three directions for the data of the University Centre in Svalbard. Regarding the meaning of the term “high-resolution” when referring to the versions of the figures included in the Open Access online dataset of the Underlying data section, it really means “full resolution”. Journals can rarely include hundreds of MB-large figures in the pdf file of articles. Hence, the versions of the figures included in the manuscript file/pdf are mostly of lower resolution than the original figures and therefore do not always allow the reader to properly view the interpreted structures (e.g., on the bathymetric and seismic data in Figure 3a–d and Figure 4a–b). The authors of the present manuscript therefore insist again on the crucial importance of both the Underlying data and the Extended data sections and agree that this is not stressed enough in the present manuscript.

Changes: Added “, including high-resolution versions of the figures and supplements, which are necessary to view the presented interpretation in detail” and “Supplementary data (e.g., calculations related to depth conversion and topographic variations, bathymetric profiles, and further field evidence and extended discussion) are available as Extended data.” to the method chapter.  

Comment 5: Also regarding methods, I feel that the results could have been bolstered by integration with the openly available gravity and magnetic data. This would allow the authors to place more constraints on the interpretations presented. Why didn’t the study utilise such potential field data? Has this work already been done?

Response: Agreed. However, the gravimetric and magnetic data mentioned by the reviewed are of too coarse resolution to be of any use in the detailed present study. The lead author of the present manuscript is currently writing a regional manuscript about the Kongsfjorden–Cowanodden fault zone, a top-SSW Timanian thrust, which extends from northwestern Russia (where it is called the Baidaratsky Fault Zone; Lopatin et al., 2001; Korago et al., 2004) and transects Spitsbergen between Sassenfjorden and Kongsfjorden and is very well imaged both by seismic data, and by gravimetric and magnetic anomalies in Svalbard (Koehl and Schiffer, in prep.).

Changes: None.  

Comment 6: I found that generally the manuscript is longer than it needs to be. Some sections in particular are much too long and the main points are lost in the text. For example, the Geological Setting section gives an excellent overview of the area, but how necessary is all of this information for the study?

Response: All the information specified in the Geological setting chapter is reused in the discussion.

Changes: None.  

Comment 7: On the other hand, the Methods section only contains two paragraphs and nowhere near enough detail is given (as described above).

Response: Agreed. However, as mentioned in earlier comments, the supplementary data ( doi.org/10.18710/1WTNQB; also found under the “Extended data” section) include extensive details about the interpretation of the stratigraphy and many more details about corresponding rocks in nearby onshore areas (see notably Supplements S1 and S5). The authors of the present manuscript concede that the importance of the Underlying data and Extended data sections should be strongly emphasized in the method chapter. See also response to comments 1 and 4.

Changes: See response to comments 1 and 4.  

Comment 8: Also regarding these introductory sections, I felt that the scientific rationale behind the work wasn’t particularly clear, i.e. what was the purpose of the study?

Response: Agreed.

Changes: Added “The paper notably explores an alternate scenario to the post-Caledonian tectonic history of Svalbard by suggesting that all post-Caledonian contractional structures may be explained by early Cenozoic Eurekan contraction alone, thus downplaying the role of the Late Devonian Svalbardian Orogeny.” to the Introduction chapter.  

Comment 9: Similarly, the discussion and interpretation sections are also overly long in my opinion.

Response: Agreed. However, the interpretation section needs to be detailed enough in order to provide robust arguments supporting the interpretation presented, especially because of the lack of well control in the southern part of the fjord. The length of the interpretation section is partly making up for the short method chapter in that much of the reasoning associated with the interpretation of the data by the authors of the present manuscript is explained in the interpretation section instead of in the method chapter. This is done this way because of the subjectivity involved in the interpretation of seismic data, which does not fit the method chapter. Regarding the discussion, much more could have been discussed as shown by the extended discussion of the tectonic evolution of the study area included in Supplement S12. The present manuscript is the first to approach the impact of reactivated Timanian thrust systems on post-Caledonian sedimentary rocks and basins in Svalbard and therefore needs sufficient discussion of the new evidence and interpretation presented in order to fully highlight their implications.

Changes: None.  

Comment 10: Finally, I found that place names/locations are not well shown on figures for readers not familiar with the setting.

Response: Agreed. See response to comment 12.

Changes: See response to comment 12.  

Comment 11: Other points: Figure 1: Overall, this is a good and useful figure but is there a colour scale for part 1 of this figure?

Response: The basemap of the figure is the International Bathymetric Chart of the Arctic Ocean (Jakobsson et al., 2012) and the reader is referred to this publication/map for the color scale. The authors of the present manuscript are open to adding the color scale from the IBCAO of Jakobsson et al. (2012) but feel that this would needlessly overcrowd the figure with a color scale from a map widely known by Arctic scientists.

Changes: None yet.  

Comment 12: Figure 3: Why are different colour scales used for A and B but no colour scale is given for D and C? What I mean by this is that I am not sure which scale I should be reading for C and D. Also what is the highshade direction used?

Response: Agreed.

Changes: Added “. The color scale shown in ( B) and encircled in a dotted black frame is that of the easternmost portion of the fjord (dataset bounded by the dotted black lines in B). The color scale shown in ( A) applies to all areas in Figure 3A–D, except the dataset bounded by dotted black lines at Nordenbskiöldbreen in easternmost Billefjorden in ( B). The light source of the hills shading in ( A–D) is from the northeast” in the caption of Figure 3. Also modified Figure 3 to add the geological map of Dallmann et al. (2004) in onshore areas and the corresponding stratigraphic legend, enlarged locality names and added a white frame so they appear clearer in Figure 3b–d, encircled the UNIS bathymetric dataset (which is associated with a discrete color scale) with a thicker dotted black line and did so for the corresponding color scale too in Figure 3b, and added the locality name “Nordenskiöldbreen” in Figure 3b.  

Comment 13: Figure 4: A better location map is needed for the seismic data, as noted above. It is unclear to me where this line is from. The text on legend is too small on this figure. The boxes on part a are very hard to see and read too against the seismic line. Also, the interpretation process leading to the yellow horizons shown needs describing further.

Response: Agreed. See response to comments 1 and 2 regarding the better localization of the interpreted seismic data and the seismic lines displayed in Figure 4 and the supplements and regarding the discussion with the editorial team to enlarge Figure 4a–b to an entire page. Regarding the interpretation of the yellow horizons, the reviewer is referred to Supplements S1 and S5 of the Extended data section. See also response to comments 1, 3, and 7.

Changes: See also response to comments 1, 2, 3, and 7.  

Comment 14: Discussion, The Adolfbukta fault: I am not sure that papers from 1997 count as recent. Even the most 'recent' one cited (2011) is 12 years old! Despite being referred to as 'older' the personal communication is the most recent item here. 

Response: Disagreed. It is specified “relatively recent”, because these studies are more recent than the older works by Harland et al. (1974), Lamar et al. (1986), and the Russians in Pyramiden (Russian data from 1988–1993 provided by Sirotkin pers comm., 2019). The authors of the present manuscript are open to adding the year of collection of the Russian data provided by Prof. Sirotkin (1988–1993), but are uncertain if and how to do this by the standard of the journal.

Changes: None yet. Awaiting further instructions from the journal.  

Comment 15: Figure 5 (and associated text): The figures that present the model are of good quality, but I don’t feel that the text explains the model sufficiently. It isn’t that the text isn’t long enough, more that the way it is explained is quite convoluted and overly long. I suggest refining the discussion so that it is more focused.

Response: Partly agreed. However, as previously mentioned, this is the first attempt at discussing the impact of the newly identified Timanian thrust systems on post-Caledonian basins and faults, and an extensive discussion of the data, interpretation, and their implications is therefore needed. See also response to comment 9.

Changes: None.

# Reviewer report: Reviewer response for version 1

**DOI:** 10.21956/openreseurope.17210.r34347

**Published:** 2023-09-12

**Authors:** Keith Dewing

**Affiliations:** 1 Geological Survey of Canada Atlantic, Dartmouth, Nova Scotia, Canada

Comments on Devonian–Carboniferous extension and Eurekan inversion along an inherited WNW–ESE-striking fault system in Billefjorden, Svalbard by JB Koehl, L Allaart & R Noormets

 Keith Dewing, Geological Survey of Canada

 This paper explores the geology of the Billefjorden area in central Spitsbergen using a seismic line and bathymetric maps. From these data, the authors conclude that pre-existing structures controlled the location of Paleozoic normal faults, and that compression is exclusively Cenozoic in age.

 The paper has a number of issues, centered around the incredibly detailed, but poorly constrained, interpretation of bathymetric and seismic datasets. These datasets are then combined with a number of the lead author’s previous studies to make broad conclusions about the regional tectonic history. The paper is hard to follow as there is a lot of detailed discussion that is not supported by maps and diagrams.

 The main issue I have with the paper is the amount of interpretation on both the bathymetric and seismic figures but that are not well supported. For instance, the interpretation of the bathymetric maps are not supported by any information from dredged samples or drilling, and the onshore geological map is not shown. The small strike-slip faults interpreted from the apparent steps in the ridge on Fig. 2D should also show up on the surface mapping. If they don’t then maybe there’s another explanation for the steps in the submarine escarpment, like scalloping due to submarine erosion or the effects of ice? If the strike slip faults only show up where there is no actually rock observation, then it would be worrisome!

 The seismic figure 4 is even more difficult to accept without a leap of faith. Firstly, why are the two overview panels so small and the inserts so big? The stratigraphic column takes a whole page yet this figure, which is critical to the arguments made in the paper, is <1/2 page. Make 4A and 4B larger. Second, there are countless interpretations that could be made on this seismic section, especially given the lack of a drill hole in the line of the section, and the absence of cross lines. I’ve marked up the figure with many comments, but I really cannot see many of the small faults that the authors have put on the figure. Not every little gap in a reflection indicates tectonic movement – there is such a thing as poor data quality. I would hesitate to interpret anything below 1.6 seconds on this section. The figure would be easier to follow if there were another panel that showed the main stratigraphic packages with partially transparent colour blocks. Trying to follow the many colours of dotted and dashed lines is tricky. 

 So while I really support new ideas being introduced into the literature, this paper needs some big improvements. There needs to be clarifying text added to the introduction that this paper presents an alternate view of the geological history that downplays the Svalbardian Orogeny and provides an interpretation where the structures can be explained by a combination of Proterozoic-Early Devonian-Cenozoic deformation and reactivation. The authors need to be clear that there are other, much more widely accepted models and that this paper presents one possibility for another tectonic interpretation.

 The bathymetric interpretation should be tied to the surface geology by showing the geological map in conjunction with the bathymetry. If the features interpreted on the bathymetric map are not shown on the surface geology map, then some explanation needs to be given as to why these features are not visible at the surface.

 The seismic figure needs to be resized, a panel is needed that shows the stratigraphic packages clearly, so the reader isn’t hunting for various dotted lines. Many of the smaller faults are pretty questionable. I don’t think most 3D cubes get this level of interpretation! Look at the literature and use a similar density of interpretation that other seismic interpreters use. I especially question 4C – why curve the upper white dotted line up rather than connect to the brown straight along trend. That would make the two white dotted lines parallel; 4D – the grouping of three reverse faults could easily be interpreted as a small graben that offsets the strong reflections at the top of the image; 4E – how can you be certain that the dotted blue lines are all the same horizon?; 4G – I just don’t see the faults you mark, why not interpret the beds as just following through? Tie the seismic to surface geology more clearly, and explain how the stratigraphic picks are made. Is it by projecting surface geology down? Recognition of seismic facies? Wishful thinking?

 It seems like the authors’ tectonic model is driving the interpretation of the seismic to a very large degree. This leads to a house of cards effect, where the questionable interpretation is presented, which in turn is supported by reference to a lot of the author’s own (often unpublished) work. Presenting citations to abstracts and unpublished work gives unjustified credence by making it look like a statement is supported by references, but those citations are not peer-reviewed.

 Reorganize discussion to focus only on the study area, cut out the regional implications that have been discussed in many of Koehl’s other papers and need not be repeated here. The discussion is very jumpy between local and regional implications and may be better organized if it were shorter and more focused. The ratio of interpretation text to data text in this paper is lop-sided in favour of interpretation. Explain the implications within the study area first, then what any new interpretation can be used in regional interpretation. Don’t just re-iterate what you’ve already published elsewhere. What is new from the study area, does it add to or support your published models? No need to spell out your previously published models in the discussion. If what you are discussing is not in the area of Figure 1, then seriously consider deleting it. The discussion could readily be cut in half.

 I have issue with citing your own unpublished work and abstracts as a back up for your interpretation. See detailed comments and editorial changes on the marked up pdf linked.

Is the study design appropriate and does the work have academic merit?

Partly

Is the work clearly and accurately presented and does it cite the current literature?

Partly

If applicable, is the statistical analysis and its interpretation appropriate?

Not applicable

Are all the source data underlying the results available to ensure full reproducibility?

Yes

Are the conclusions drawn adequately supported by the results?

No

Are sufficient details of methods and analysis provided to allow replication by others?

Partly

Reviewer Expertise:

Arctic tectonics; stratigraphy; petroleum systems

I confirm that I have read this submission and believe that I have an appropriate level of expertise to state that I do not consider it to be of an acceptable scientific standard, for reasons outlined above.

# Response

**Published:** 2023-10-14

**Authors:** Jean-Baptiste Koehl

**Affiliations:** Geosciences, Universitetet i Oslo, Oslo, Oslo, Norway

**Reply to Keith Dewing**   Dear Sir, Madam, thank you very much for your input on the manuscript, it is highly appreciated. Here is our reply to your comments. We hope the changes we implemented improve the shortcomings of the manuscript highlighted by your comments and suggestions. Please do not hesitate to contact us shall this not be the case for some comments.  

**Comments by the reviewer**

Comment 1: This paper explores the geology of the Billefjorden area in central Spitsbergen using a seismic line and bathymetric maps. From these data, the authors conclude that pre-existing structures controlled the location of Paleozoic normal faults, and that compression is exclusively Cenozoic in age.

Response: Mostly agreed, although the authors of the present manuscript would like to add that they argue that most of the post-Caledonian contraction is Cenozoic in age. In addition to Cenozoic Eurekan contractional deformation, the authors of the present manuscript recognize Timanian contraction and acknowledge the widespread occurrence of Caledonian contraction in the area.

Changes: None.  

Comment 2: The paper has a number of issues, centered around the incredibly detailed, but poorly constrained, interpretation of bathymetric and seismic datasets. These datasets are then combined with a number of the lead author’s previous studies to make broad conclusions about the regional tectonic history. The paper is hard to follow as there is a lot of detailed discussion that is not supported by maps and diagrams.

Response: Disagreed. From this comment and other comments by the reviewer, it is clear that he has not had a look at the Extended data and Underlying data sections available open access on DataverseNO ( https://doi.org/10.18710/1WTNQB and https://doi.org/10.18710/UCRW4L respectively). It is therefore crucial that these sections are further highlighted and the reader better encouraged to look there early on. See response to comments 4, 5, 6, 7, 8, 10, 11, 12, 13, 14, 15, 16, 17, 65, and 67, and the “Other changes” section at the end of the present document.

Changes: Added “, including high-resolution versions of the figures and supplements, which are necessary to view the presented interpretation in detail” and “Supplementary data (e.g., calculations related to depth conversion and topographic variations, bathymetric profiles, and further field evidence and extended discussion) are available as Extended data.” in the method chapter. See response to comments 4, 5, 6, 7, 8, 10, 11, 12, 13, 14, 15, 16, 17 65, and 67, and the “Other changes” section at the end of the present document.  

Comment 3: The main issue I have with the paper is the amount of interpretation on both the bathymetric and seismic figures but that are not well supported. For instance, the interpretation of the bathymetric maps are not supported by any information from dredged samples or drilling, and the onshore geological map is not shown.

Response: No drilling and no dredging were ever done in the fjord. Therefore, the authors of the present manuscript have to proceed without such data.

Changes: None.  

Comment 4: The small strike-slip faults interpreted from the apparent steps in the ridge on Fig. 2D should also show up on the surface mapping. If they don’t then maybe there’s another explanation for the steps in the submarine escarpment, like scalloping due to submarine erosion or the effects of ice? If the strike slip faults only show up where there is no actually rock observation, then it would be worrisome!

Response: The small strike-slip faults depicted by the offset of the ridge on bathymetric data in the fjord do indeed show on adjacent onshore areas on the western shore of the fjord. For this, the reader is referred to the supplementary data attached to the present manuscript. Notably supplements S10 and S11 show evidence for WNW–ESE-striking faults onshore Billefjorden on satellite images (S10) and in the field (S11) at Narveneset and Brimerpynten (see Figures 1b and 3d for location). Furthermore, supplement S11 clearly documents sinistral strike-slip kinematics along these faults in the field, thus supporting that the interpreted apparent offset of the N–S- to NNW–SSE-striking ridge in the fjord is at least partly related to sinistral strike-slip tectonic fault offset.

Changes: None required.  

Comment 5: The seismic figure 4 is even more difficult to accept without a leap of faith. Firstly, why are the two overview panels so small and the inserts so big? The stratigraphic column takes a whole page yet this figure, which is critical to the arguments made in the paper, is <1/2 page. Make 4A and 4B larger.

Response: The format the journal chose to display the figure may hopefully be adapted after discussion with the editorial team.

Changes: Awaiting further discussion with the editorial team to enlarge figure 4a–b potentially to an entire page.  

Comment 6: Second, there are countless interpretations that could be made on this seismic section, especially given the lack of a drill hole in the line of the section, and the absence of cross lines. I’ve Open Research Europe Page 26 of 29 Open Research Europe 2023, 3:124 Last updated: 22 SEP 2023 marked up the figure with many comments, but I really cannot see many of the small faults that the authors have put on the figure.

Response: The authors of the present manuscript concede that it is not possible to identify all the small faults interpreted in Figure 4a–b in the pdf file of the manuscript. However, the authors of the present manuscript made both supplementary data (see Extended data section at the end of the present manuscript; https://doi.org/10.18710/1WTNQB) and underlying data (see Underlying data section at the end of the present manuscript; https://doi.org/10.18710/UCRW4L) available so that the readers may fully zoom in and out of each figure, exploring the interpretation presented with full resolution. The high-resolution versions of the figures of the present manuscript are several hundreds of MB to several GB large and therefore cannot be included as such in the manuscript’s pdf file. Nevertheless, the authors of the present manuscript argue that the high-resolution versions of the figures provide ample opportunity to the reader to zoom in specific structures on the seismic and bathymetric data.

Changes: None.  

Comment 7: Not every little gap in a reflection indicates tectonic movement – there is such a thing as poor data quality. I would hesitate to interpret anything below 1.6 seconds on this section.

Response: Partly agreed. However, the seismic data presented in Figure 4a–b is of relatively high quality for the area and shows an incredible wealth of tectonic structures. This specific seismic line does not show any of the typical seismic artifacts (e.g., multiples, diffraction rays), that are often observed on poor-quality seismic data in the area (e.g., supplement S3 and S4 in which the authors of the present manuscript clearly avoided interpreting any reflection past 0.6 seconds TWT because of the poor quality of the data and the clear artifacts present on the data, e.g., multiples). Again, the reader is referred to the Underlying data section of the present manuscript, which is fully accessible on DataverseNO (Open Access online data repository; https://doi.org/10.18710/UCRW4L).

Changes: None.  

Comment 8: The figure would be easier to follow if there were another panel that showed the main stratigraphic packages with partially transparent colour blocks. Trying to follow the many colours of dotted and dashed lines is tricky.

Response: Agreed.

Changes: Redesigned Figure 4b to include colored polygons for each stratigraphic unit.  

Comment 9: So while I really support new ideas being introduced into the literature, this paper needs some big improvements. There needs to be clarifying text added to the introduction that this paper presents an alternate view of the geological history that downplays the Svalbardian Orogeny and provides an interpretation where the structures can be explained by a combination of ProterozoicEarly Devonian-Cenozoic deformation and reactivation. The authors need to be clear that there are other, much more widely accepted models and that this paper presents one possibility for another tectonic interpretation.

Response: Agreed.

Changes: Added “The paper notably explores an alternate scenario to the post-Caledonian tectonic history of Svalbard by suggesting that all post-Caledonian contractional structures may be explained by early Cenozoic Eurekan contraction alone, thus downplaying the role of the Late Devonian Svalbardian Orogeny.”.  

Comment 10: The bathymetric interpretation should be tied to the surface geology by showing the geological map in conjunction with the bathymetry. If the features interpreted on the bathymetric map are not shown on the surface geology map, then some explanation needs to be given as to why these features are not visible at the surface.

Response: Agreed.

Changes: Added the geological map by Dallmann et al. (2004) in figure 3a, c, and d in the present manuscript, as well as the corresponding legend. Regardless of this change, very little can be extracted from the onshore geological map because it is way too detailed and will anyway require the reader/reviewer to refer to the map by Dallmann et al. (2004) and zoom in and out to identify specific stratigraphic units amongst the numerous units in the area.  

Comment 11: The seismic figure needs to be resized, a panel is needed that shows the stratigraphic packages clearly, so the reader isn’t hunting for various dotted lines.

Response: Agreed. See response to comments 5 and 8.

Changes: See response to comments 5 and 8.  

Comment 12: Many of the smaller faults are pretty questionable. I don’t think most 3D cubes get this level of interpretation! Look at the literature and use a similar density of interpretation that other seismic interpreters use.

Response: The present manuscript is not about doing seismic interpretation the same way many scientists have done in the past, but about advancing geological research by using the cutting-edge, ground-breaking leap made by the first authors in his correlation of geological structures in the field to their equivalent on seismic reflection data, e.g., Koehl (2020, 2021) and Koehl et al. (2022a, 2023). In these contributions, the lead author of the present manuscript notably describes a correlation of S-shaped and Z-shaped seismic reflection data to onshore duplex structures, and the geometry of hundreds of meters wide asymmetric (verging, recumbent, isoclinal) folds and mylonitic shear zones and thrusts. This research was commended by various scientists in the field and was recognized by an invitation as a Keynote speaker at the EGU 2020 (Koehl, 2020). As to whether 3D data can get us this far, the answer is yes, absolutely, but if and only if one has trained one’s eyes to hunt for specific types of reflections and reflection patterns (e.g., Koehl et al., 2023b). Arguing that seismic data are of poor quality is no longer a valid excuse in the case of Figure 4a–b because we now have tools and potential explanations for most of the reflections and seismic facies displayed in the seismic line.

Changes: Added reference to Koehl et al. (2023) in the present manuscript.  

Comment 13: I especially question 4C – why curve the upper white dotted line up rather than connect to the brown straight along trend. That would make the two white dotted lines parallel;

Response: Disagreed. This would be inconsistent with the negative amplitude reflection (blue) located just above the interpreted dotted white line, which clearly curves up. Therefore, it is more probable that the positive amplitude reflection (red) also does the same at this specific location.

Changes: None.  

Comment 14: 4D – the grouping of three reverse faults could easily be interpreted as a small graben that offsets the strong reflections at the top of the image;

Response: Disagreed. By calling it a “graben” the reviewer is suggesting that there is a south-dipping normal fault disrupting the positive amplitude reflection (red) above the dotted black (negative amplitude) reflection. However, the reflection interpreted as a dotted black line and all underlying reflection are not disrupted, therefore invalidating the suggested interpretation of a graben. A much more likely alternative is that the disruption of the positive amplitude (red) reflection above the dotted black line is offset by a small north-dipping thrust, which accommodated top-south movement and is dying out above and below the offset reflection because no further offsets are observed. Note that this is typical in sedimentary successions with strong rheological contrasts between interbedded units such as rocks of the Carboniferous–Permian Gipsdalen Group in Billefjorden, which typically consists of weak evaporites and shales interbedded with strong sandstone–siltstone and carbonates. In the present case, the most likely candidate are carbonate- and evaporite-rich rocks of the Wordiekammen Formation and/or Gipshuken Formation (Gee et al., 1952; Cutbill & Challinor, 1965; Keilen, 1992; Ahlborn & Stemmerik, 2015).

Changes: Added small thrust mentioned here in Figure 4d.  

Comment 15: 4E – how can you be certain that the dotted blue lines are all the same horizon?;

Response: Just like every single seismic interpreter, the authors of the present manuscript do not pretend to be sure of anything. However, the very similar seismic amplitude and geometry of the reflector throughout the area support the correlation presented. Note that the reviewer and any other geoscientist may very well come up with another interpretation of the presented data. Up to present, the interpretation proposed in the present manuscript is a first ever presented.

Changes: None.  

Comment 16: 4G – I just don’t see the faults you mark, why not interpret the beds as just following through?

Response: Partly agreed. The top red line interpreted by the reviewer may very well represent a continuous bed, although it is a strong oversimplification by the reviewer considering the undulating geometry of the reflection’s northern half. The undulating geometry is not left our in the present interpretation and the authors of the present manuscript argue that it is related to top-south thrust faults arranged in imbricates. This interpretation gains weight when looking at the reflections below the top red line interpreted by the reviewer, which are more undulating and/or disrupted, thus forming packages of Z-shaped reflections (dotted white lines). Such reflections are typical in weak sedimentary successions deformed during contractional events, especially in the study area (e.g., Koehl, 2021). The alternate interpretation of the reviewer, though simplifying considerably the overall interpretation and geology of the area, undermines several key features and therefore misses the overall picture.

Changes: None.  

Comment 17: Tie the seismic to surface geology more clearly, and explain how the stratigraphic picks are made. Is it by projecting surface geology down? Recognition of seismic facies? Wishful thinking?

Response: Again, from the reviewer’s present comment, it is clear that he has not read/had access to the Supplementary data mentioned in the Extended data section in the present manuscript and available open access on DataverseNO (open access online data repository; https://doi.org/10.18710/1WTNQB). The reviewer is invited to review carefully Supplements S1 (named “Description of the late Paleozoic sedimentary successions in central Spitsbergen from the literature”) and S5 (entitled “Description and interpretation of the upper Paleozoic sedimentary successions on seismic data in Billefjorden”). Among others, the stratigraphic pics on the seismic section were made considering the onshore geology of Dallmann et al. (2004) and many other studies in the study area (e.g., Harland et al., 1974; Cutbill et al., 1976; Ringset and Andresen, 1988; Smyrak-Sikora et al., 2018), as well as the known rock types in each stratigraphic units and their potential expression (seismic facies) on seismic data (see Supplements S1 and S5 in the Extended data section for an extended description of each relevant stratigraphic units and their correlation to seismic facies on the interpreted seismic data). The authors of the present manuscript regret that they did not make it clearer that the Extended data (Supplements S1 to S14; https://doi.org/10.18710/1WTNQB) and the Underlying data (i.e., high-resolution versions of the manuscript’s figures and supplements; https://doi.org/10.18710/UCRW4L) were paramount in evaluating the quality of the work done. See also response to comment 2 for changes implemented to address this need.

Changes: See response to comment 2 and Supplements S1 and S5 for more information on the information required by the reviewer’s comment.  

Comment 18: It seems like the authors’ tectonic model is driving the interpretation of the seismic to a very large degree. This leads to a house of cards effect, where the questionable interpretation is presented, which in turn is supported by reference to a lot of the author’s own (often unpublished) work. Presenting citations to abstracts and unpublished work gives unjustified credence by making it look like a statement is supported by references, but those citations are not peer-reviewed.

Response: The reviewer is referred to Supplements S1 and S5 of the Extended data section ( https://doi.org/10.18710/1WTNQB) for further information on how the interpretation of the stratigraphic units was done and on the arguments used to correlate onshore geology to seismic facies. The authors of the present manuscript would like to remind the reader that it is because of the strong resistance to change of many experienced/senior geologists, who generally get selected for peer-review work, that the work by young researchers gets published ever so slowly if it ever gets published. It is therefore necessary to take the appropriate steps so that the work by early-career researchers is not forgotten, which sometimes means publishing the work without it passing the peer-reviewing process. This happens regularly for small scientist communities such as the Geosciences, especially for areas with very few groups and individuals working in (e.g., Svalbard). In addition, most scientists having a different opinion than the main published models are generally in either one of the following two situations: (1) they do not have a permanent position and therefore would like to wait until they secure a permanent position before they share their new ideas with the rest of the world and irritate/anger experienced scientists who might not agree with them, or (2) they finally obtained a permanent position and wonder why they should bother “fighting” and trying to convince their peers and should not simply relax, focus on minor issues that are unlikely to arise emotion or anger anyone at all, and enjoy the ride instead of arguing and create tension with others who might very well be colleagues at the same institute or friends (or family). As human beings, we are naturally biased and unobjective, and oftentimes lack the courage to be disliked because of the need of the feeling that one belongs to a specific community and contributes to that community. The authors of the present manuscript argue that the present interpretation is the result of a large review of all the database of seismic data available in and around Svalbard and the northern Barents Sea, together with gravimetric and magnetic data, all the bathymetric data in and around Svalbard, field data and geochronological and microstructural data. The present interpretation is one of the only interpretations that reconcile all the datasets in the area. Note that a careful review and interpretation of all the datasets mentioned was done before any of the manuscripts written by the lead author of the present manuscript were written, i.e., the interpretation presented in the present manuscript is not driven by the model published in the other studies by the lead author of the present manuscript, but is rather part of a huge work, which would have never been possible to publish as a single manuscript (see also response to comment 50).

Changes: None. See also response to comment 50.  

Comment 19: Reorganize discussion to focus only on the study area, cut out the regional implications that have been discussed in many of Koehl’s other papers and need not be repeated here. The discussion is very jumpy between local and regional implications and may be better organized if it were shorter and more focused. The ratio of interpretation text to data text in this paper is lop-sided in favour of interpretation. Explain the implications within the study area first, then what any new interpretation can be used in regional interpretation. Don’t just re-iterate what you’ve already published elsewhere. What is new from the study area, does it add to or support your published models? No need to spell out your previously published models in the discussion. If what you are discussing is not in the area of Figure 1, then seriously consider deleting it. The discussion could readily be cut in half.

Response: Partly agreed. This is exactly what the present discussion is trying to do: show that the findings in the present manuscript are in agreement with the interpretation of Timanian thrust systems throughout Svalbard (Koehl, 2020; Koehl et al., 2022a) and with the non-occurrence of the Svalbardian Orogeny in Svalbard (Koehl et al., 2022c). In addition, most of the areas discussed are in Svalbard or adjacent to Svalbard (e.g., Timanian thrust systems in Storfjorden). See response to comments 62, 63, 64, and 65.

Changes: See response to comments 62, 63, 64, and 65.  

Comment 20: I have issue with citing your own unpublished work and abstracts as a back up for your interpretation. See detailed comments and editorial changes on the marked up pdf linked.

Response: Disagreed. The reviewer seems to have an issue with the non-peer-reviewed work of the lead author of the present manuscript rather than the “unpublished” work. Let it show for the record that all the work cited in the present manuscript is published, although not all works cited are peer-reviewed, and that absolutely all the studies (both peer-reviewed and non-peer-reviewed) by the lead author of the present manuscript are available online at open access repositories and/or on ResearchGate. Nevertheless, the cited work that is not yet peer-reviewed includes clear photographs of the described outcrops and interpreted structures in the field and are linked to Open Access datasets including extensive outcrop photographs and structural measurements (all available on DataverseNO). Comparing the studies done by the lead author of the present manuscript to the studies done by previous workers who established the occurrence of the Svalbardian Orogeny in Svalbard, a significant difference is the absence of any field photograph in the latter. The Svalbardian event was first proposed by T. Vogt, most of whose work is unavailable and possibly completely lost because never digitalized. The next group of workers to strongly argue in favor of and set the foundation of the Svalbardian Orogeny in Svalbard is the group of K. Piepjohn in the 90s. It is important to note that there rarely are any strata overlying tectonically deformed Devonian strata in Svalbard, thus rendering constraining the timing of the observed deformation in Devonian sedimentary rocks difficult. However, in the very few places that might provide crucial insights, e.g., Alvrekkdalen, Asvindalen, Tordalen, Munindalen, and Mimerdalen in central Spitsbergen near Billefjorden, none of the studies by the group of K. Piepjohn shows any field photograph (Piepjohn et al., 1997; Piepjohn, 2000; Dallmann and Piepjohn, 2020). Instead, these studies include exclusively idealized sketches drawn by the authors themselves on the basis of monodisciplinary scientific approach (structural geology) and monodisciplinary data (structural fieldwork), or sometimes on long-distance observation of large transects largely eroded and mostly covered by screes and/or inaccessible for detailed inspection because located on steep slopes and cliffs, which the authors of the present manuscript find astounding. The quality of the interdisciplinary research and the robustness of the conclusions drawn by the authors of the present manuscript can therefore not be compared with that of the group of Dr. K. Piepjohn. It is also worth noting that Dr. K. Piepjohn and his group tried pining the Svalbardian Orogeny in Svalbard based on highly questionable (and this is a massive understatement) paleontological evidence (Piepjohn et al., 2000), which included only one specimen of misidentified Retispora lepidophyta (see discussion in Koehl et al. 2022c and in Berry and Marshall, 2015 their supplement DR3), the sample of which can no longer be located (K. Hartkopf-Froeder pers. comm., 2020).

Changes: See “Other changes” section at the end of the present document.  

Comment 21: deleted “The present contribution is part of a large study (Koehl et al., 2020) aiming at investigating cryptic WNW–ESE-striking structures and fabrics in the Norwegian Arctic.”

Response: Disagreed. This sentence was specifically requested by the journal’s editorial team.

Changes: None.  

Comment 22: Maybe be clear that this paper presents an alternate view of the geological history that downplays the Svalbardian Orogeny and provides an interpretation where the structures can be explained by a combination of Proterozoic-Early Devonian-Cenozoic deformation and reactivation.

Response: See response to comment 9.

Changes: See response to comment 9.  

Comment 23: Not sure truncated is clear in this context. Clarify what was truncated? Or cut by or was part of?

Response: Agreed.

Changes: Added “Precambrian rocks of” and changed “was” into “were”.  

Comment 24: What is CC-BY4?

Response: CC-BY4 is an Open Access type of license. Please refer to the Creative Commons webpage for more information.

Changes: None requested.  

Comment 25: Earyl.

Response: Agreed.

Changes: Updated to “Early”.  

Comment 26: Nothing in the Mesozoic? There are Triassic-Jurassic strata on your map.

Response: That is correct. Not every strata is deposited during a period of active tectonism. The Mesozoic in Svalbard is generally thought to have been deposited during tectonic quiescence. Since these strata and geological periods are irrelevant to the present study, it is certainly best to leave them out of an already long manuscript.

Changes: None.  

Comment 27: The.

Response: Agreed.

Changes: Added “at”.  

Comment 28: Glacier. Might help for those of us who struggle with all these geographic names.

Response: Although adding “glacier” here would indeed help the reader grasp the nature of the locality described, it would also be redundant. In order to be consistent, one would need to change the name from “Nordenskiöldbreen” to “Nordenskiöld glacier”, which is not entirely optimal, especially if one is to search the Norwegian Polar Institute’s database of localities around the Svalbard Archipelago (toposvalbard.npolar.no): one would not find any entry under “Nordenskiöld glacier”. However, entering “Nordenskiöldbreen would immediately zoom in the specified area. In addition, the nature of the specified locality (glacial) is irrelevant to the present study, so it does not matter if the locality name is after a glacier, a valley or anything else. What matters is its actual location, which can be obtained in Figure 1 or from the toposvalbard.npolar.no database.

Changes: None.  

Comment 29: Delete “gentle to moderate (typically” and “)”.

Response: Agreed.

Changes: Deleted “gentle to moderate (typically” and “)”.  

Comment 30: Is the vertical scale time or thickness?

Response: None.

Changes: None.  

Comment 31: Why do you only see jogs related to the strike slip in the offshore (where there is no hard data) but not onshore where the geology is exposed? Why not show the onshore geology on this map too?

Response: The geology is not exposed everywhere, especially in the investigated, deeply eroded Arctic areas where outcrops are typically of very poor quality with low vertical and lateral continuity, and/or located on steep cliffs and slopes and therefore inaccessible for detailed inspection. In addition, the investigated offshore area in southern Billefjorden (Figure 3d in the present manuscript) provides a laterally continuous horizontal/map view of the structures in the area. By contrast, outcrops in adjacent onshore areas are neither flat, nor vertical, but display outcrop curvature varying both in dip angle and orientation. Therefore, the jogs observed offshore would not be jogs onshore and might even be partly eroded. The along-strike variations in overall kinematics, geometry, and units it juxtaposes have been and continue to be a major issue when studying the onshore exposures of the Billefjorden Fault Zone as pointed out in Koehl (2021, 4 ^th^ paragraph pp. 1041). It is time to stop considering onshore field data as “hard data” or so to say data that are more robust than offshore and/or subsurface data Onshore outcrops do indeed include their share of scientific uncertainty related to the interpretation scientists make of them. In addition, the hundreds to thousands of kilometers horizontal and several to tens of kilometers vertical continuity of, e.g., of good-quality seismic data is way higher than that of onshore outcrops, which generally show vertical continuity in the order of a few tens of meters and horizontal continuity in the order of a few hundreds of meters if lucky (which is rarely the case anywhere in the study area; see also the datasets of outcrop photographs published by the lead author of the present manuscript in the study area at DataverseNO: e.g., Koehl and Stokmo, 2021 https://doi.org/10.18710/BIJYVO; Koehl et al., 2022 https://doi.org/10.18710/NARMZS). Furthermore, offsets related to strike-slip (i.e., horizontal) movements are better observed in map view, i.e., in the setting observed in the offshore portion of the fjord in southern Billefjorden (Figure 3d in the present manuscript), whereas onshore outcrops would hardly show such well expressed offset due to extensive and differential fluvial and glacial erosion in the area. See also response to comments 3, 4, and 10

Changes: See response to comments 3, 4, and 10.  

Comment 32: Hard to see what this fault is offsetting.

Response: If the reviewer is referring to the third southernmost WNW–ESE-striking fault in Figure 3d in the present manuscript: Not all the WNW–ESE-striking faults need to show some lateral offset. The fault pointed out by the reviewer may very well show little to no lateral offset, or that lateral offset may not be apparent possibly because of glacial erosion. If the reviewer is referring to the fourth southernmost WNW–ESE-striking fault in Figure 3d in the present manuscript: that fault offsets laterally the onshore Gipshuken Fault (Harland et al., 1974) from its offshore continuation (Balliolbreen Fault) in the fjord.

Changes: Added “and its southern continuation, the Gipshuken Fault” and “Harland et al., 1974”.  

Comment 33: Hard to see a fault here too.

Response: Agreed, and this is why the associated line is stippled. However, the onshore geology suggests the presence of the northeast-dipping Cowantoppen Fault (Harland et al., 1974) at the present location, whose onshore expression correlates with the stippled, WNW–ESE-striking submarine escarpment.

Changes: Added “An example is the southernmost WNW–ESE-striking escarpment in Billefjorden ( Figure 3D), which correlates with the onshore occurrence of the northeast-dipping Cowantoppen Fault (Harland et al., 1974)” to the interpretation section of the bathymetric data.  

Comment 34: You can map these through the moraine?

Response: If there is a pronounced trend of WNW–ESE-striking faults in Billefjorden, it is possible that some of these are actually newly formed Cenozoic (Quaternary?) faults forming due to minor adjustments in the crust and therefore partly cut through the moraine. This would explain the slightly oblique character of smooth, glacial WNW–ESE-striking lineations and WNW–ESE-striking fault-related escarpments in Figure 3b in the present manuscript. This is supported by recent earthquakes and ongoing movement/activity along most of the major Timanian thrust systems mapped by Koehl et al. (2022a) in Storfjorden and southern Svalbard, which are responsible for ≥ 75% of all Mw ≥ 4.0 earthquakes in the past 100 years and controlled the formation of small, shallow, WNW–ESE-striking brittle faults, which crosscuts the seafloor in Storfjorden (Koehl and Rimando, 2023 submitted; see also Pirli et al., 2010, 2013). Analogously, N–S-striking faults such as the Billefjorden Fault Zone controlled the formation of recent landslides in Billefjorden (e.g., Munindalen, Odellfjellet, and Garmaksla; Dallmann et al., 2004; Koehl et al., 2022 submitted).

Changes: None.  

Comment 35: Is the note about copyright needed? Is it separate from the copyright associated with this article?

Response: This was specifically requested by the journal.

Changes: None.  

Comment 36: I would not be comfortable interpreting below 1.6 seconds.

Response: See response to comments 5, 6, 7, 11, and 12.

Changes: See response to comments 5, 6, 7, 11, and 12.  

Comment 37: Just for fun – why not join these blue reflections up? It seems just as viable an interpretation, but cuts across most of your interpreted structures.

Response: Agreed, and this interpretation might very well be valid as a structure, e.g., as a top-south (mylonitic?) Eurekan thrust. However, it is not optimal if interpreted as a stratigraphic reflection from a seismic facies’ perspective in that it would not fully separate reflections with large amounts of steep disruptions interpreted as Proterozoic basement rocks in the present manuscript. A potential interpretation other than a top-south Eurekan thrust might be an intra-basement lithological boundary. As it stands now, the northern half of the reflection is disrupted from the southern half, and it is possible that the northern half actually represents a multiple of the overlying reflection interpreted as a clear Top-basement erosional unconformity (pink line) with downlaps (white half arrows) in overlying (Lower Devonian) sedimentary strata. The two reflections show the exact same geometry and the time distance between them is comparable to that of sea level and the seafloor (Figure 4a–b in the present manuscript). The authors of the present manuscript are open to include the reflection highlighted by the reviewer in the present manuscript’s interpretation provided that the reviewer provides further support as to why the highlighted northern half of the suggested reflection should not be a multiple.

Changes: None. Awaiting further arguments from the reviewer.  

Comment 38: This is A LOT of interpretation considering the quality of the data an lack of drill control or cross lines. Most 3D cubes aren’t getting this level of detail. This figure should be much larger and the insets much smaller.

Response: See response to comments 5, 6, 11, and 12.

Changes: See response to comments 5, 6, 11, and 12.  

Comment 39: How does the compression get to these tiny reverse faults? Where is the decollement, why aren’t the big red normal faults taking up the movement instead?

Response: The area consists of strong basement rocks tightly deformed during at least two major episodes of contraction orthogonal to one another: NNE–SSW-oriented Timanian contraction and E–W-oriented Caledonian contraction. Then, Devonian–Carboniferous collapse-related extension kicked in and resulted in the formation of moderate to high-angle normal faults (see also supplement S7 for depth-converted version of Figure 4a–b). Such high-angle normal faults were simply not suitable to accommodate more than minor reverse movement shown as gentle open folding. Instead, it was much easier to create new low-angle faults and deform the very weak, shale-rich Devonian succession, which acted as a buffer between very strong basement rocks and relatively strong sandstone–siltstone- and carbonate-rich Carboniferous–Permian sedimentary strata.

Changes: None.  

Comment 40: Why not bring upper white dotted line here?

Response: See response to comment 13.

Changes: See response to comment 13.  

Comment 41: I cant see this as a primary stratigraphic structure?

Response: Agreed. And it is not suggested by the present interpretation. The white half-arrow simply pointed at the termination of a seismic reflection, seemingly truncated upwards. The authors of the present manuscript would like to point out that many similar reflections are highlighted in Figure 4b in the present manuscript and that the upwards truncation of the highlighted seismic reflections are mostly tectonic in nature (e.g., due to arrangement into contractional duplexes or thrust imbrication). Please note that only some of the reflections highlighted by white half-arrows are interpreted as sedimentary onlaps, toplaps and downlaps (e.g., in Figure 4d).

Changes: None.  

Comment 42: The offset on this structure don’t make a lot of sense.

Response: The offsets on the highlighted structure make perfect sense: (1) formation of a bedding-parallel décollement along the weak blue marker/bed, (2) offsetting of the décollement by low-angle thrusts and staking of the blue bed/marker, and (3) formation of high-angle reverse faults (or inversion of preexisting Devonian normal faults) crosscutting the low-angle thrusts and décollement, possibly because the thrust stack became locked due to tremendous amount of lateral movement.

Changes: Added “showing (1) a possible bedding-parallel décollement (dotted blue line), (2) low-angle thrusts offsetting and staking sheets of the décollement onto one another, and (3) high-angle reverse faults (or inverted preexisting Devonian normal faults) crosscutting the low-angle thrusts and décollement, possibly because the thrust stack became locked due to tremendous amount of lateral movement” in the caption of Figure 4e.  

Comment 43: I cant see these faults on 4A – I suggest that the lines I ve draw in red would work better.

Response: Disagreed. See response to comment 16.

Changes: See response to comment 16.  

Comment 44: This fault doesn’t seem to offset anything?

Response: Disagreed. If the reviewer’s comment is targeting one of the northern two early Cenozoic thrusts (yellow lines), these offset two levels of Z-shaped reflections/beds (dotted white lines). At both levels, the thrusts offset the Z-shaped reflections from one another. If the reviewer is targeting the inverted Devonian normal fault (red line), the fault appears to die out upwards, but it clearly offsets many reflections below the orange-marked reflection, where each reflection is juxtaposed against a reflection of opposite polarity.

Changes: None.  

Comment 45: What is the evidence for these?

Response: The evidence for these is a continuous reflection (dotted dark green line) capping disrupted and upwards terminating reflections. Onshore, evidence for these are tightly folded and thrust, weak Lower Devonian rocks (mostly shales) of the Wood Bay Formation below thick, strong carbonate beds of the Wordiekammen Formation observed in many occurrences and localities in the study area, which decoupling at or near the stratigraphic unconformity between the two formations. This notably occurs in Asvindalen (just southwest of Brimerpynten), Brimerpynten, Narveneset (location in Figure 3 in the present manuscript), Garmdalen (just north of Narveneset), Yggdrasilkampen, and Reuterskiöldfjellet (in Mimerdalen; see location at toposvalbard.npolar.no). Importantly, and although the onshore geology part of the this work is not yet published, this claim is indeed possible to verify by checking the outcrop photographs of the lead author of the present manuscript by visiting the related dataset on DataverseNO (Koehl and Stokmo, 2021 https://doi.org/10.18710/BIJYVO, days 0, 2, 4, 5, and 8 in the dataset; note that overview photographs of the Reuterskiöldfjellet locality are also available from the other days of the field trip, specifically days 6 and 7). The reviewer is notably referred to photographs IMG_3779 to 3797 in the folder “Eirik”, which show the tectonized unconformity between the Devonian and Permian with bedding-parallel gouge (photo IMG_3797) and embrittled, sigma-clast-looking blocks in the Devonian indicating both top-west and top-east transport direction within tens of cm from one another (i.e., extremely limited movement, probably in the range of a few meters). The authors of the present manuscript are open to mentioning the onshore evidence in the Extended data supplement available on DataverseNO should this be judged necessary. However, they would prefer to do so in the upcoming manuscript.

Changes: None. Awaiting decision by the reviewer and the editors.  

Comment 46: Not dashed on figure?

Response: Disagreed. This line is both dashed (in the hanging wall of the Garmaksla fault, just below the thick dashed red line) and not dashed (everywhere else) in Figure 4b in the present manuscript.

Changes: None.  

Comment 47: It was submitted but never accepted? I wouldn’t cite something that is uploaded by yourself on ResearchGate as scientific literature. Peer review is here for a reason – it does prevent some crazy ideas from gaining traction.

Response: The authors of the present manuscript wonder what the more trustworthy is between (1) a peer-reviewed fieldwork-based study including zero outcrop photograph and no access to the structural data used (i.e., no access to any of the data used in the study to evaluate their claim; e.g., Piepjohn et al., 1997; Piepjohn, 2000), which was peer-reviewed by a couple of reviewers, and (2) a non-peer-reviewed manuscript published open access (e.g., Koehl et al., 2022 submitted; dx.doi.org/10.13140/RG.2.2.28031.33448) including actual outcrop photographs, which are linked to supplements including high-resolution versions of each photograph and figure as supplements (Supplements S1 to S11), and linked to datasets of structural data and numerous outcrop photographs in the study area all available open access on DataverseNO ( doi.org/10.18710/BIJYVO and doi.org/10.18710/TIIIKX), so that everyone (i.e., not only a couple of favorable reviewers) may review and evaluate their claim?

 According to the FAIR research principles, the latter is ethically correct and appropriate to be submitted for peer review, whereas the former is not. The manuscript in question here (Koehl et al., 2022 submitted) was submitted and rejected unfairly by unobjective reviews by a couple of disgruntled senior scientists. The lead author of the present manuscript and of the study in question has a clean conscience and all the evidence used in the rejected manuscript is fully presented and available to the reader in the manuscript, in the supplements attached to the manuscript (S1 to S11), and at the online open access data repository DataverseNO ( doi.org/10.18710/BIJYVO and doi.org/10.18710/TIIIKX). The goal of the manuscript in question is to discredit old-fashioned, monodisciplinary work previously done in the study area (Billefjorden) by specific geologists who ended up being selected to review the manuscript.

 The lead author of the present manuscript and of the study in question regrets that he had the naivety to believe that these two peer-reviewers would produce objective reviews and behave in a manner fit to a senior researcher. It is now obvious that these senior scientists did not want to let their models (which they seem to view as their legacy) go down without an unethical pushback, i.e., an unobjective review. Peer review is certainly here for many reasons, but sometimes used by senior scientists to retain control over what new ideas are being published, which is in a certain way unfair, because younger scientists with more updated/modern ways of proceeding (both ethically – cf. FAIR research principles, and scientifically – e.g., interdisciplinary approach) generally end up being rejected (and segregated when applying for positions in academia) if they disagree with models previously published by senior researchers. Unfortunately for these senior scientists and fortunately for early-career scientists, knowledge is like water. If there is a crack in the hull, the ship will inevitably capsize, i.e., erroneous geological models will eventually be abandoned, albeit much later than they could have, therefore significantly delaying scientific progress. Note that such a scenario is rendered unlikely by the open, author-driven peer-review system created by Open Access Research. The authors of the present manuscript would like to call the attention of the readers and reviewers of the present work to the fact that the present study is part of an earlier manuscript, which included the present manuscript and the following two (now published and peer-reviewed) articles: Koehl et al. (2023a) and Koehl et al. (2023c). This earlier large manuscript was rejected by senior reviewers who lacked the skill (notably in interpreting bathymetric and seismic data) to appreciate the merit and impact of the work that had been done and/or lacked the courage to be disliked and allow new ideas opposing commonly accepted models (including their own models) to be published. Consequently, tremendous time, human, and financial resources have been wasted to divide the earlier large manuscript into three discrete manuscripts (this issue will be addressed by the lead author of the present manuscript in a manuscript about science ethics and the peer review process in the Geosciences). This pitfall occurs very commonly in the Geoscience community and appropriate steps should be taken to avoid this to continue to happen. In the end, is it not more important that a “crazy” idea goes through the peer review process and is available for the taking and for pursuing in the event it eventually turns out that it is the most realistic model (e.g., the idea of Continental Drift by Wegener and du Toit), or that a crazy idea is rejected and never published and therefore never pollutes the mind of new researchers nor becomes available to future researchers in dire need of new solutions to reconcile all the new data that become available with the dawn of the Open Access movement? Some “crazy” ideas far less realistic than the idea presented in the present manuscript because monodisciplinary and requiring a much more complicated model with more tectonic movements have previously been accepted by the peer review process (e.g., the 2000-km-sinistral-strike-slip-displacement model for the Great Glen Fault based exclusively on paleomagnetic data; van der Voo and Scotese, 1981), and these ideas have now been fairly abandoned. However, the fact that these ideas were allowed to be published in the first place allowed a broader community to further test them. If an idea/product is so crazy, e.g., destroying one’s health with heavy drugs, has it evet helped that the local authorities in charge ban/veto the idea/product? Is it not when an idea or product is banned by the authorities in charge (in the present case, the more experienced scientists selected as peer reviewers) that it actually gains even more attention? In the end, each and every individual will be confronted with the choice to either give in the new idea or resist it.

Changes: None.  

Comment 48: These are not helpful in the interpretation?

Response: Yes, the dashed white lines representing intra-basement reflections are indeed important for the present interpretation because they highlight the obliquity of potential stratigraphic intra-basement reflections and mylonitic surfaces within the main shear zone in the south. This relationship is highlighted in the first paragraph of the “Structures in Proterozoic basement rocks” section.

Changes: None.  

Comment 49: Im thinking about the relative weights of evidence for glacial vs resistant bed as part of the stratigraphic package vs resistant unit brought up by faulting. It might be helpful to have the onshore geology shown next to the bathymetric maps? This would allow the reader to see how close the correspondence is between the submarine features and mapped onshore features.

Response: Agreed. See response to comment 10.

Changes: See response to comment 10.  

Comment 50: There is far more interpretation on the seismic than I would be willing to make. There must be hundreds of ways to interpret this line! How much of the interpretation is being driven by the tectonic model that the authors wish to display? The lack of drill control for checks on age and velocity structure leads to a decrease in confidence. There is all sorts of noise that comes in from

Response: Some of the seismic artifacts mentioned by the reviewer were taken into account by the authors of the present manuscript (see response to comment 37). See also response to comment 4. The interpretation presented in the present manuscript is based on several field campaigns by the lead author in the Billefjorden area between 2015 and 2021, including visits of onshore areas directly adjacent to the seismic line in Narveneset and Brimerpynten (see also Extended data and Underlying data sections in the present manuscript and the attached datasets https://doi.org/10.18710/1WTNQB and https://doi.org/10.18710/UCRW4L). Although there must indeed be hundreds of ways to interpret every single seismic section ever acquired (including those with one or more well controls), the authors of the present manuscript have provided scientific arguments as to why they believe that the presented interpretation is likely the correct one (cf. description section of the stratigraphic units on seismic data and corresponding units in the Geological setting chapter and the extended description in supplement S1 in the Extended data, including notably detailed arguments on the correlation of rock types and rock type variation with seismic facies; https://doi.org/10.18710/1WTNQB). Despite the suggestion by the reviewer that the presented interpretation is driven by earlier interpretation by the lead author of the present manuscript published in previous articles (e.g., Koehl et al., 2022a, 2023b), the data presented herein were interpreted before the lead author came to the conclusion that the only way to reconcile all the data in the entire Barents Sea, northern Norway, Svalbard, and the Fram Strait was that of continuous Timanian thrust systems, which were repeatedly reactivated and overprinted during the Caledonian Orogeny, Devonian–Carboniferous extensional collapse, Eurekan contraction, and late Cenozoic rifting (which then led him to write several manuscripts on the matter, including Koehl et al., 2022a, 2023b). If it had been up to the lead author of the present manuscript, he would have happily submitted his ground-breaking discovery of continuous Timanian thrust systems as a large monograph or book including the entire database (including field data, seismic, bathymetric, magnetic, gravimetric, well bore, and geochronological data) he interpreted between 2016 and 2020 in the whole Barents Sea, northern Norway, Svalbard, and the Fram Strait. Unfortunately, because of the current peer-review system in place, if the lead author of the present manuscript had attempted to compile his whole work into a book/monograph, not only it would have taken him 2–3 years of focused work doing just that in a world driven by a “publish or perish” attitude (in which one has to find funding for research and for salary, teach, and keep developing one’s skills and CV to hopefully get a permanent position someday, and in which one also has to face constant nation-wide bullying because proposing a new idea diverging from previously published models), but in addition to this he would have had to deal with obtuse reviewers who may have abandoned their dignity and rejected flat out his work (which the lead author of the present manuscript has unfortunately witnessed already many times in his career although still an early-career scientist). Again, the present work and the two related manuscripts (Koehl et al., 2023a, 2023c) are a textbook illustration of this pitfall of the current peer-review system: the three manuscript were initially submitted as a whole ( http://dx.doi.org/10.13140/RG.2.2.35857.97129), but the senior scientists tasked to evaluate the work failed to see its merit, and/or unobjectively disagreed with its conclusions, and/or did not have all the necessary skills to fully judge this interdisciplinary piece of work (especially when it comes down to seismic interpretation, which the lead author is an expert of with no less than 11 courses in seismic interpretation during his Masters Degree and two years in the hydrocarbon industry). The reviewers both agreed that in order to be published, this work ( http://dx.doi.org/10.13140/RG.2.2.35857.97129) had to be split into 2 to 3 separate manuscripts (#forcedsalamislicing), which is now almost done and took over 3 years. The lead author of the present manuscript regrets that the current peer-reviewing system does not allow smoother publishing of ground-breaking discoveries because of the unreasonable amount of power given to senior researchers (by comparison to early-career researchers) who commonly keep doing research at the same level and using the same tools as at the time of their Ph.D. during their whole career because there is no time to learn new skills in our constantly busy modern work environments. The author of the present manuscript takes the opportunity to salute the new, author-driven, open peer-review system offered by the journal Open Research Europe, which is much fairer because a manuscript cannot be rejected because of one or two unobjective review. Instead, the manuscript will still be out there, published (though not yet passed peer-review) and the authors will be able to keep improving it and to keep suggesting more appropriate reviewers.

Changes: See response to comment 4.  

Comment 51: Are they mylonitic onshore?

Response: Yes, some onshore faults in basement rocks are mylonitic (Koehl et al., 2023c their figure 4d and e).

Changes: None required.  

Comment 52: As interpreted on.

Response: Agreed that the content of the parenthesis is confusing.

Changes: Deleted “and” and moved “Koehl *et al*., 2023b their figures 2–6” to a different parenthesis earlier in the same sentence.  

Comment 53: Spelling.

Response: Agreed.

Changes: Updated “modetarely” into “moderately”.  

Comment 54: The former are interpreted to have been deposited as an alluvial fan.

Response: Agreed.

Changes: Replaced “were” by “are interpreted to have been”.  

Comment 55: Im having a hard time seeing these.

Response: See response to comments 5 and 6.

Changes: See response to comments 5 and 6.  

Comment 56: I would have interpreted the folds at the top of 4D as a little graben created by normal faults rather than a compressional feature. This is an example of where the tectonic model drives the interpretation. Maybe Im not looking hard enough on 4B, but where is the folding in the top Billefjorden Grp?

Response: One thing is to have a model drive an interpretation and another is to produce an interpretation that reconciles all the datasets and observations (i.e., not only those presented in the present manuscript, but also all over Svalbard, the Barents Sea, northern Norway, and the Fram Strait). In the present case, the latter approach was used. The Top Billefjorden Group reflection (dotted green line) is indeed potentially mildly folded (see curving geometry of the reflection), but this reflection only appears on a small portion of the seismic line, so if the authors of the present manuscript had to consider only this reflection alone, their interpretation would be inconclusive. Nevertheless, this is what is argued for here: that the synclinal geometry of strata of the Ebbadalen (i.e., Lower Pennsylvanian) and Minkinfjellet formations although they are mildly folded partly reflects the syn-kinematic character of the Ebbadalen Formation, whereas the Minkinfjellet Formation is late-tectonic or postdates normal faulting. It is the interpretation of the character of all the reflections that determined the interpretation of the seismic line. See also response to comment 14.

Changes: See also response to comment 14.  

Comment 57: Can be attributed to?

Response: Agreed.

Changes: Changed “are imputable” into “can be attributed”.  

Comment 58: This whole paragraph seems peripheral to the current work. Haakon VII Land doesnt even show on the map, so the reader cant follow this thread easily.

Response: Agreed that the location of Haakon VII Land is missing in Figure 1a. However, the present paragraph is highly relevant to the present discussion because it is about analogous findings in an adjacent area of Spitsbergen, compares them to the study area, and provides perspective for recent studies of potential metamorphic core complex-related detachments (e.g., Braathen et al., 2018).

Changes: Added the location of Haakon VII Land in Figure 1a.  

Comment 59: Deleted “the occurrence of”.

Response: Agreed.

Changes: Delete “the occurrence of”.  

Comment 60: This text is unclear to me. The features that link hard and soft need to be pointed out.

Response: Agreed that the highlighted text may be confusing.

Changes: Added “(two thick, deep yellow lines in Figure 4B)” and “(two thick, shallow yellow lines in Figure 4B)”, and split the sentence into two and rewrote the second sentence into “The shallow and deep thrustsa re separated by aggregates of top-SSW contractional duplexes ( Figure 4A–B), which may correspond to soft-linked portions of the thrusts”.  

Comment 61: Is this sentence needed?

Response: Yes, it is. It refers the reader to an important piece of work that demonstrates that the Svalbardian Orogeny presents severe issues. The authors of the present manuscript firmly oppose the idea of the Svalbardian Orogeny in Svalbard, but the authors of the present manuscript do not oppose the publishing of this idea nor its discussion by its believers.

Changes: None.  

Comment 62: A lot of this paragraph seems out of scope of the paper. This is general Svalbard, not just the study area. Haven't these ideas already been published in Koehl and Munoz-Barrera etc. Why do they need to be restated here?

Response: Disagreed. The present paragraph compares WNW–ESE-striking faults in western Spitsbergen to the identified WNW–ESE-striking faults in the study area. These ideas have not been published in Koehl and Muñoz-Barrera (2018). The authors of the present manuscript believe that it is important for the reader to note that the interpretation argued for in the present manuscript has very strong similarities with the WNW–ESE-trending segment of the West Spitsbergen Fold-and-Thrust belt in western Spitsbergen, and that similar Eurekan structures occur in the same stratigraphic units (e.g., Gipshuken and Wordiekammen formations), which show very comparable lithological variations as in Billefjorden both onshore and offshore. In addition, the present paragraph sets the stage for paragraphs 2 and 3 of the “Possible origin for WNW–ESE-striking faults and shear zones in Billefjorden” section, which suggest that the WNW–ESE-striking faults and shear zones in the study area are part of a major late Neoproterozoic Timanian thrust system, which controlled the formation of subsequent, early Cenozoic Eurekan thrusts both in Billefjorden (study area) and in western Spitsbergen.

Changes: None.  

Comment 63: Again, this paragraph is expanding the results of this study to the broad region. Seems like an over-reach.

Response: Disagreed. The present paragraph is needed to highlight the high intensity of Eurekan deformation in central Spitsbergen (i.e., some distance away from the Eurekan front in western Spitsbergen) and show that the alternate interpretation proposed for the study area in central Spitsbergen is also highly relevant for areas in western Spitsbergen (i.e., near the Eurekan front or within the West Spitsbergen Fold-and-Thrust Belt), where previous studies postulated (i.e., no geochronological constraints) a Late Devonian age for numerous thrust structures in pre-Carboniferous rocks (e.g., Thiedig and Manby, 1992; Kempe et al., 1997). Nonetheless, the authors of the present manuscript concede that the present paragraph is poorly organized.

Changes: Reorganized and split the paragraph into two, and added “(especially in the west near the West Spisbergen Fold-and-Thrust Belt)” and “Therefore, differences in deformation type and intensity between basement, Devonian, and post-Devonian strata may be explained by Eurekan tectonism alone” to the newly created paragraph.  

Comment 64: All this is in other publications?

Response: The present paragraph highlights the similarity of structures (e.g., bedding-parallel décollements) in a nearby area (Sassenfjorden) to those investigated in the study area (Billefjorden). It is therefore argued that the present paragraph is needed to give further weight to the presently proposed interpretation.

Changes: None.  

Comment 65: Hard to follow without detailed maps. Again, this seems to be fighting a bigger battle than is warranted by the data and interpretation presented.

Response: Disagreed. If the reviewer’s comment targets the upper paragraph, it is about the Billefjorden area (i.e., the study area of the present manuscript). If the reviewer’s comment targets the lower paragraph, it is also about Billefjorden (i.e., the study area of the present manuscript). Regarding the reviewer’s comment on the need for a detailed map, it is available in the Figure 1b. However, it is still not enough to fully appreciate the topography, which is available in the detailed map by Dallmann et al. (2004) and at svalbardkartet.npolar.no (see also response to comment 67). Note that the calculations, the topographic data needed, and the source of the data is available in Supplement S12 of the Extended data (available on DataverseNO at https://doi.org/10.18710/1WTNQB).

Changes: See also response to comment 67.  

Comment 66: ??

Response: Specifically requested by the journal.

Changes: None.  

Comment 67: No data presented to help the reader follow along.

Response: Disagreed. The data dealt with include topographic data (specifically the altitude at which rocks of certain stratigraphic units crop out onshore) available in Dallmann et al. (2004) and at svalbardkartet.npolar.no (including both topographic data and the geological map), and offshore depth conversion available on DataverseNO (Supplement S6 in Extended data available at https://doi.org/10.18710/1WTNQB and a high-resolution version of the table at https://doi.org/10.18710/UCRW4L). Note that the calculations, the topographic data needed, and the source of the data is available in Supplement S12 of the Extended data (available on DataverseNO at https://doi.org/10.18710/1WTNQB).

Changes: None.  

Comment 68: Delete.

Response: Disagreed. This would undermine the impact of the presentation.

Changes: None.  

**Other changes: **Updated Koehl et al. (2023), Koehl et al. (2023 submitted), Koehl et al. (2023b), and Koehl et al. (2023c) into Koehl et al. (2023a), Koehl et al. (2023b), Koehl et al. (2023c), and Koehl et al. (2023d) respectively due to the recent publishing of Koehl et al. (2023 submitted).

# Reviewer report: Reviewer response for version 1

**DOI:** 10.21956/openreseurope.17210.r34344

**Published:** 2023-08-25

**Authors:** Liang Qiu

**Affiliations:** 1 State Key Laboratory of Geological Processes and Mineral Resources, School of Earth Sciences and Resources, China University of Geosciences, Beijing, China

The manuscript demonstrates a commendable overall organization and introduces a novel dataset of seismic reflection profiles. The figures are clear and the text is generally well-crafted. Herein, I bring to your attention a few minor points of consideration.

 The article delves into the Cenozoic and the Devonian to Carboniferous periods; however, it also provides an introduction to the geological setting of the Neoproterozoic and early Paleozoic eras. Consequently, the initial two paragraphs could be omitted or simplified.

 It seems that the research significance in the section of Introduction appears to be relatively straightforward and superficial, lacking sufficient depth.

 Certain sentences within the main text could benefit from further refinement, such as the following examples: ‘The present contribution is part of a large study (Koehl *et al.,* 2020) aiming at investigating cryptic WNW–ESE-striking structures and fabrics in the Norwegian Arctic. The present contribution focuses on the offshore portion (seismic and bathymetric data) of the Billefjorden area, whereas Koehl *et al. *(2023a) and Koehl *et al.* (2023b) focus on onshore outcrops respectively on the western shore and the eastern shore of the fjord.’

 Fig.1 lacks latitude and longitude.

 The figure panels referenced in the text are presented in uppercase, such as Figure 3A, while all figure numbers are rendered in lowercase.

 The legend of Figure 4 is too tiny to see.

 Koehl *et al., *2022a or Koehl *et al.,* 2022b?

Is the study design appropriate and does the work have academic merit?

Yes

Is the work clearly and accurately presented and does it cite the current literature?

Yes

If applicable, is the statistical analysis and its interpretation appropriate?

Not applicable

Are all the source data underlying the results available to ensure full reproducibility?

Yes

Are the conclusions drawn adequately supported by the results?

Partly

Are sufficient details of methods and analysis provided to allow replication by others?

Yes

Reviewer Expertise:

Structural geology and tectonics; geochronology

I confirm that I have read this submission and believe that I have an appropriate level of expertise to confirm that it is of an acceptable scientific standard.

# Response

**Published:** 2023-10-14

**Authors:** Jean-Baptiste Koehl

**Affiliations:** Geosciences, Universitetet i Oslo, Oslo, Oslo, Norway

**Reply to Liang Qiu**   Dear Prof. Qiu, thank you very much for your input on the manuscript, it is highly appreciated. Here is our reply to your comments. We hope the changes we implemented improve the shortcomings of the manuscript highlighted by your comments and suggestions. Please do not hesitate to contact us shall this not be the case for some comments.  

**Comments by the reviewer**

Comment 1: The article delves into the Cenozoic and the Devonian to Carboniferous periods; however, it also provides an introduction to the geological setting of the Neoproterozoic and early Paleozoic eras. Consequently, the initial two paragraphs could be omitted or simplified.

Response: The first two paragraphs are rather simple and non-extensive and are absolutely needed to introduce the episodes of deformation that are discussed in the Discussion chapter. If any, the authors of the present manuscript are open to merging the two paragraphs or to expand them further, although more details are already given in the discussion.

Changes: None yet.  

Comment 2: It seems that the research significance in the section of Introduction appears to be relatively straightforward and superficial, lacking sufficient depth.

Response: Agreed.

Changes: Add the following sentence at the end of the first paragraph of the Introduction chapter: “In addition, the N–S-striking structural trend does neither explain the provenance of Lower Devonian sediments of the Wood Bay Formation from the south-southwest in northern Spitsbergen (Friend and Moody-Stuart, 1972), nor the sourcing of the Central Tertiary Basin from the north-northeast in the Paleocene (Petersen et al., 2016).”. The authors of the present manuscript also recommend the addition of the following paragraph at the end of the Introduction chapter: “The present study has implications for the late Neoproterozoic–Phanerozoic tectonic evolution of the Svalbard Archipelago and for plate tectonics modelling, which typically place the accretion of Svalbard’s basement terrane in the mid-Paleozoic through large lateral tectonic movements along hundreds to thousands of kilometers long, N–S-striking fault zones (e.g., Billefjorden Fault Zone). The structures discussed in the present contribution provide new anchor points for the Svalbard Archipelago with the Barents Sea block and Baltica in the late Neoproterozoic and introduce new limitations on the possible amount of N–S-oriented lateral tectonic movements. The present work therefore calls for major revisions of current local plate tectonics models for Arctic regions and full-plate models, notably regarding large-scale lateral movements of blocks and terranes during short periods of time, which is not possible to reconcile with the longevity and continuous character of inherited Neoproterozoic structures. Furthermore, the present results show the influence of strain partitioning on deformation is discrete stratigraphic units, thus underlining the value of interdisciplinary studies over monodisciplinary (e.g., structural) approaches. Finally, the present study shows that the Billefjorden Trough is not a typical rift basin as generally suggested by previous studies in the area.”.  

Comment 3: Certain sentences within the main text could benefit from further refinement, such as the following examples: ‘The present contribution is part of a large study (Koehl et al., 2020) aiming at investigating cryptic WNW–ESE-striking structures and fabrics in the Norwegian Arctic. The present contribution focuses on the offshore portion (seismic and bathymetric data) of the Billefjorden area, whereas Koehl et al. (2023a) and Koehl et al. (2023b) focus on onshore outcrops respectively on the western shore and the eastern shore of the fjord.’

Response: These sentences were included on specific request by the editorial team. They must therefore remain unchanged.

Changes: None.  

Comment 4: Fig.1 lacks latitude and longitude.

Response: Agreed.

Changes: Added latitude and longitude to Figure 1a.  

Comment 5: The figure panels referenced in the text are presented in uppercase, such as Figure 3A, while all figure numbers are rendered in lowercase.

Response: Agreed. The authors of the present manuscript are open to updating the figure numbers according to the reviewer’s suggestion pending that it is in line with the journal’s format.

Changes: Awaiting decision by the editorial team.  

Comment 6: The legend of Figure 4 is too tiny to see.

Response: Agreed. The authors of the present manuscript recommend the journal to separate figure 4a–b from figure 4c–h and to expand figure 4a–b to an entire page to allow the readers to view the presented interpretation in detail, pending that it is alright with the journal.

Changes: Awaiting decision by the editorial team.  

Comment 7: Koehl et al., 2022a or Koehl et al., 2022b?

Response: Both Koehl et al. (2022a) and Koehl et al. (2022b) are listed in the reference list.

Changes: None required.
